# Supplementary material for: Spontaneous whole-genome duplication restores fertility in interspecific hybrids
Source: Nat Commun. 2019 Sep 11;10:4126. doi: 10.1038/s41467-019-12041-8 (PMC6739354; doi:10.1038/s41467-019-12041-8)
Supplement: Supplementary file 1 — Supplementary Information [file 41467_2019_12041_MOESM1_ESM.pdf]

Supplementary Information

Spontaneous whole-genome duplication restores fertility in interspecific hybrids

Charron, Marsit, *et al*.

This PDF file includes:

Supplementary Notes 1 and 2
Supplementary Figures 1 to 22
Supplementary Tables 1 to 8
Supplementary References (1-8)

Supplementary Notes

Supplementary Note 1

To investigate the potential mechanisms of whole genome doubling that led to tetraploid hybrids we sequenced the genome of all tetraploids at T_{ini} and T_{end}. The main way by which autotetraploidization could occur by mating in our study would have been to have a mating between two hybrids with damage to the opposite *MAT* loci. This implies damages to the *MAT* loci but also that the two cells with damaged loci are in close proximity such that they could mate. Such tetraploid hybrids should thus have two *MAT* loci of opposite mating type with damaged function. By combining read coverage and allele frequency analysis, we investigated the presence of total or partial loss of chromosome III, which contains the *MAT* locus (Supplementary Figures 18 and 19) or loss of heterozygosity (LOH) events around the *MAT* locus (Supplementary Figure 20). We identified two tetraploids that show aneuploidy on chromosome III at T_{end}. One of the L1 tetraploids has one additional copy of chromosome III from the *SpC* parent and the H2 tetraploid has lost one copy from the *S. cerevisiae* parent. In both cases, aneuploidy affects only one copy of the mating type. Considering the fact that total or partial loss of chromosome III should affect the two mating type loci in the tetraploid hybrid, these results show that the loss of chromosome III is not the molecular mechanism leading to whole genome doubling. Allele frequency analysis confirms these aneuploidies and show that there is no LOH events around the *MAT* locus in all tetraploids (Supplementary figure 20).

We also examined the copy number of *MAT*a and *MAT*α sequences from each parent (Supplementary figure 21). For an accurate analysis, we had to take in account the silent mating type loci. Indeed, in addition to the *MAT* locus, *Saccharomyces* yeasts carry two unexpressed, but complete, copies of mating-type genes at the silent loci, *HML* and *HMR,* which are localised also on chromosome III (Supplementary Figure 20)[1]. The *HML* locus carries *MAT*α sequence while *HMR* carries *MAT*a sequence. Thus, knowing that the *SpB* parent is *MAT*a and the *SpC*, *SpA* and *S. cerevisiae* parents are *MAT*α, each hybrid carries both mating type copies from each parent with a different copy number of *MAT*a or *MAT*α corresponding to each parental origin.

Copy number and allele frequency variation observed in *MAT*a/*MAT*α copies from each parent (Supplementary Figure 21) are due either to the aneuploidies mentioned above (L1_51) (Supplementary Figure 18), to an LOH in the *HML* locus (L2_36) (Supplementary Figure 20) or

to a combined effect of aneuploidy and LOH in the *HML* locus (H2_38) (Supplementary Figures 18, 20 and 21), which could not have led to mating.

Our results show no evidence of damages at the *MAT* locus in tetraploid hybrids that could have caused mating between diploid hybrids. The cases of aneuploidies or LOH observed affect only one parental copy of the *MAT* locus or the silent *HML* locus. Also, there is no evidence for damaging mutations occurring in the *MAT* loci. Thus, according to these results, autodiploidization is the most likely mechanism of whole genome doubling

Supplementary Note 2

Genome sequencing allowed us to examine the genome of the M1 tetraploid (M1_40) whose ploidy and fertility increased at T_{mid} and then decreased at T_{end}. This result could be explained by the presence of heterogenous colonies during our evolution experiment. The isolated colony at T_{mid} may have contained segregating diploid and tetraploid cells. One type may have fixed in the glycerol stock and be lost in the next round of propagation, explaining why we observe only tetraploids at T_{mid}. However, the colony isolated during the subsequent passage was a diploid one, explaining why we observe only diploids in subsequent passages.

Besides, GBS data are consistent with ploidy and fertility data for M1_40 line, however genome sequencing shows a triploid state at T_{end} instead of diploid. The detection of chromosome I loss of the *SpA* parent copy at T_{ini} and T_{end} (Supplementary Figure 18) confirms that both sequenced isolated colonies correspond to the same M1_40 line at two different times. The same variation is observed for the M2_36 line at T_{ini} that show a different state between ploidy and GBS data (2n) and sequencing data (4n, with many aneuploidies) while the same LOH is observed in chromosome VIII at T_{ini} and T_{end} (Supplementary Figure 16). These results suggest that there is still heterogeneity among isolated colonies from the glycerol stock, probably due to high genomic instability of hybrids.

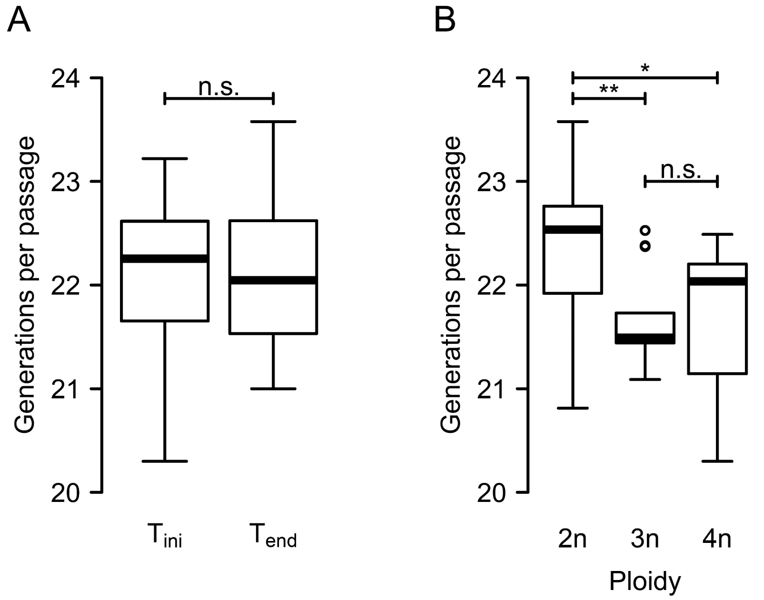

Supplementary Figure 1. The number of mitotic generations per passage is stable through time but differs between hybrids with different ploidies.

Number of mitotic generations estimated as the log_2 of the number of cells in colonies after a growth cycle by following the same experimental procedure as for the evolution experiment. This was performed for a subset of 40 lines. The number of cells was estimated by flow cytometry of a colony resuspended in water. (**A**) There is no statistically significant difference in the number of mitotic generations when considering the timepoints (T_{ini} or T_{end}) of the experiment (Welch two sample t-test, $P = 0.902$). **(B)** There is a significant effect of ploidy (one-way ANOVA ($F(2,60) = 9.256$, Tukey HSD, "**" $P < 0.01$, "*" $P < 0.05$, "n.s." non-significant), showing that changes to higher ploidy were not favored by selection during the experiment. For all boxplots, the bold center line corresponds to the median value, the box boundaries correspond to the 25th and the 75th percentile, the whiskers correspond to 1.5 times the inter-quartile range and the dots to outlier values. Source data are provided as a Source Data file.

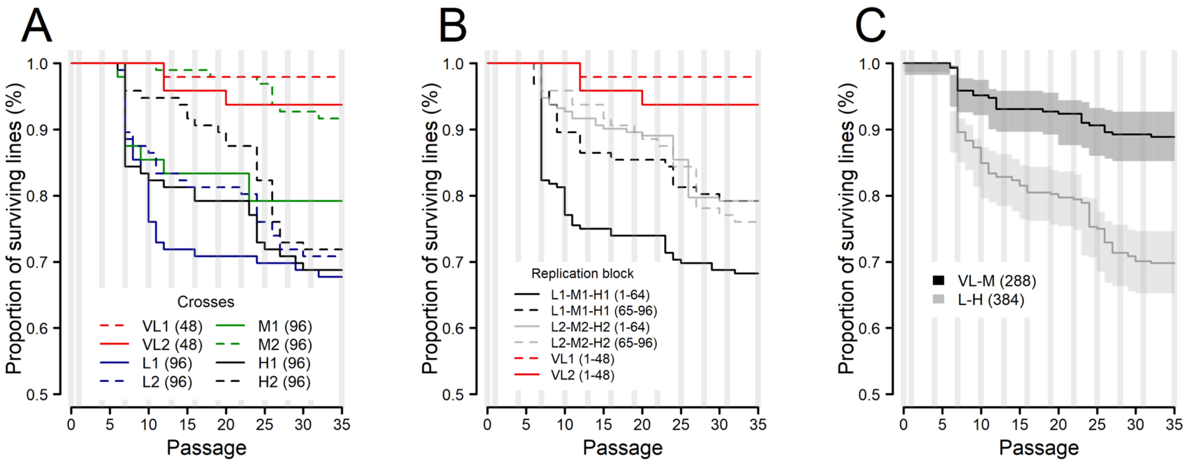

Supplementary Figure 2. Survival of the evolution lines.

Survival of the lines grouped by (**A**) individual crosses, (**B**) replication sets used during the evolution experiment and (**C**) crosses showing high (VL-M) and low (L-H) survival. Source data are provided as a Source Data file.

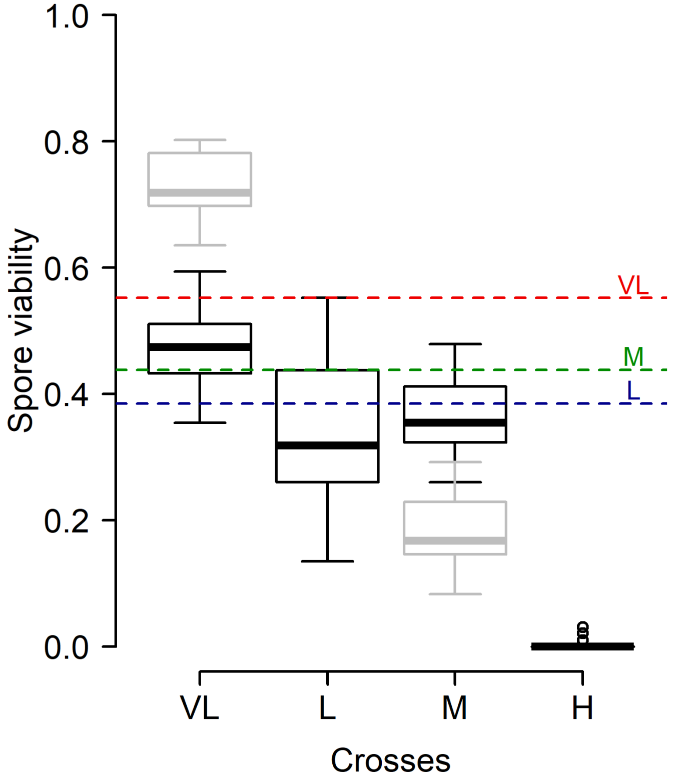

Supplementary Figure 3. Ancestral lines show expected spore viabilities for their cross types. Boxplot of spore viabilities for the different hybrid lines at T_{ini}. A single box represents the average for the two crosses when their mean is not different. In case of significant difference in mean, black and grey boxes represent the first and second crosses respectively. Dashed colored lines represent median spore viabilities values for each of the cross types from Leducq *et al*. (2016)[2]. For all boxplots the bold center line corresponds to the median value, the box boundaries correspond to the 25th and the 75th percentile, the whiskers correspond to 1.5 times the inter-quartile range and the dots to outlier values. Source data are provided as a Source Data file.

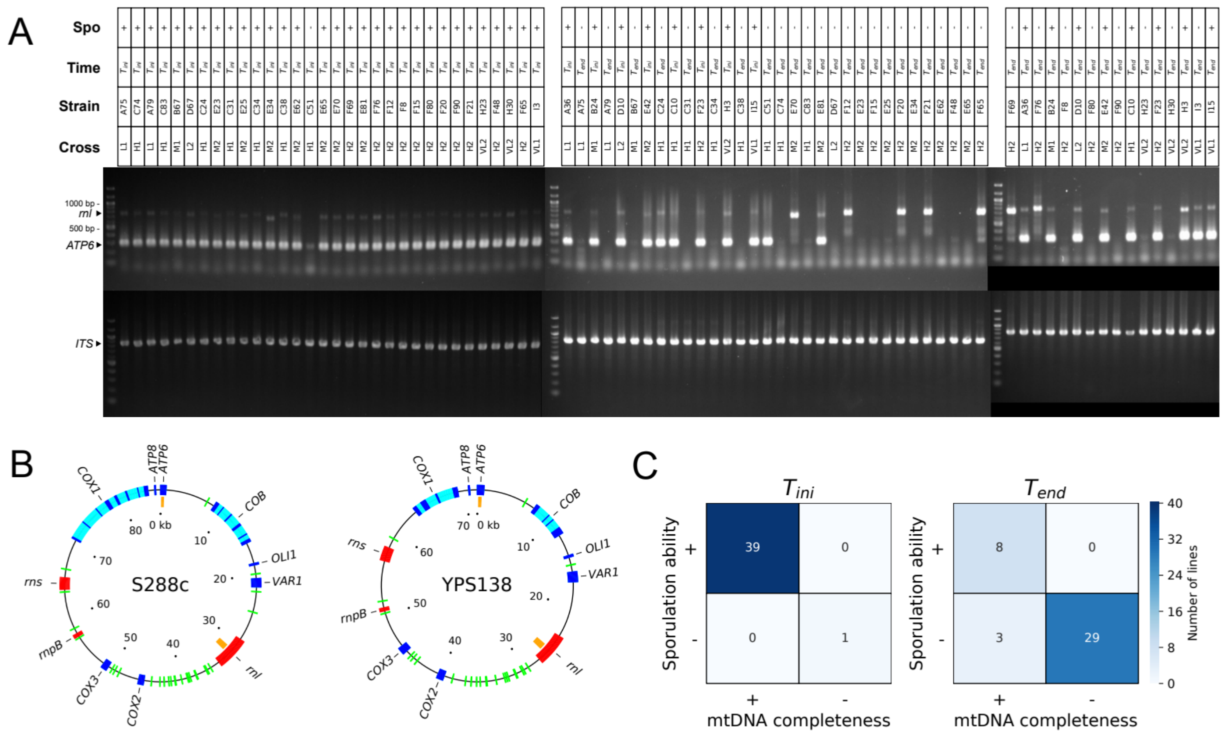

Supplementary Figure 4. Partial or complete mtDNA deletion is associated with the loss of sporulation ability after 770 mitotic generations. (A) PCR assays for the *rnl* and *ATP6* mitochondrial loci and for the *ITS* nuclear locus. Presence or absence of these loci was assayed for 32 lines which lost the ability to sporulate at the end of the mitotic evolution experiment and 8 lines that maintained the ability. PCRs were performed on DNA extracted from stocks from T_{ini} and T_{end} timepoints. The *rnl* amplicon is expected to vary in size around 700 bp, while the *ATP6* amplicon is expected to be 285 bp long. (**B**) Map of *S. cerevisiae* (S288c) and *S. paradoxus* (YPS138) mtDNAs. The *rnl* and *ATP6* amplicons are shown with orange shapes. Protein-coding genes are shown in blue, protein-coding gene introns in cyan, RNA-coding genes in red and tRNAs in green. The genome annotations used are from Yue et al. 2017 [3]. (**C**) Partial or complete loss of mtDNA is associated with loss of sporulation ability. For T_{ini} and T_{end}, contingency tables show the counts of lines according to mtDNA completeness (+: both markers present, -: at least one marker absent) versus sporulation capacity. The association is significant for T_{end} (Fisher's exact test, odds ratio > 77, P-value=1.43×10^{-5}).

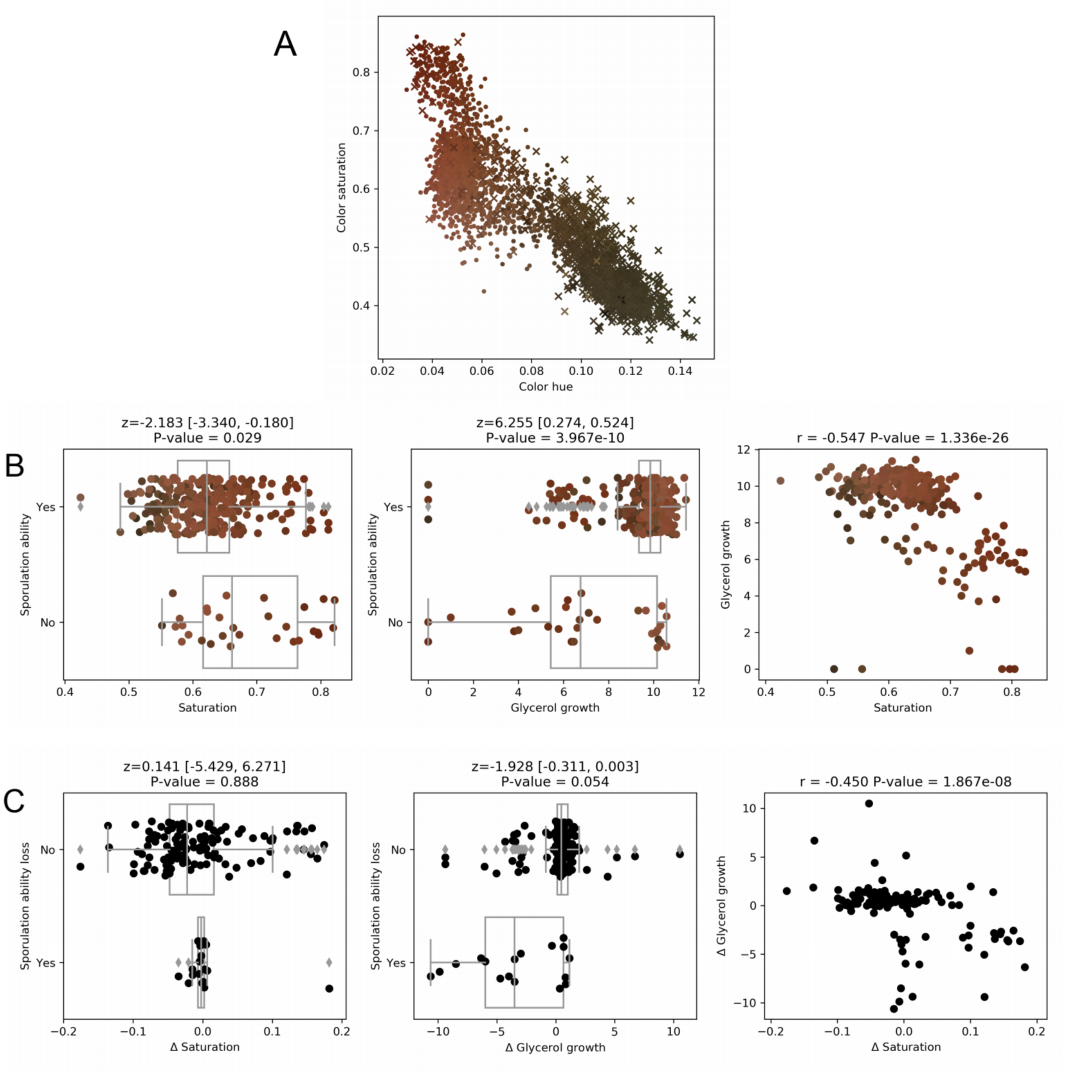

Supplementary Figure 5. Sporulation inability is associated with increase in red pigment saturation and decrease of growth on glycerol. (A) Raw data of the colony image analysis shows that color hue discriminates between background and colonies, while color saturation captures the variation in red pigmentation intensity. Dots: data points kept for the analysis, cross symbols: data points filtered out (see Methods). **(B)** Sporulation ability is significantly associated with color saturation (left) and growth on glycerol (center). P-values and 95% confidence intervals for logistic regressions are shown. Saturation and growth on glycerol are correlated (right). P-value for Pearson's correlation is shown. Boxes show the 1st, 2nd and 3rd quartiles of the data, while whiskers show data points within 1.5 inter-quartile range. **(C)** Sporulation ability loss with time shows no significant association with change in color saturation (left) and a marginally significant association with change in growth on glycerol (center). P-values and 95% confidence intervals for logistic regression are shown. Variations of saturation and growth on glycerol are correlated (right). P-value for Pearson's correlation is shown. Boxes show the 1st, 2nd and 3rd quartiles of the data, while whiskers show data points within 1.5 inter-quartile range.

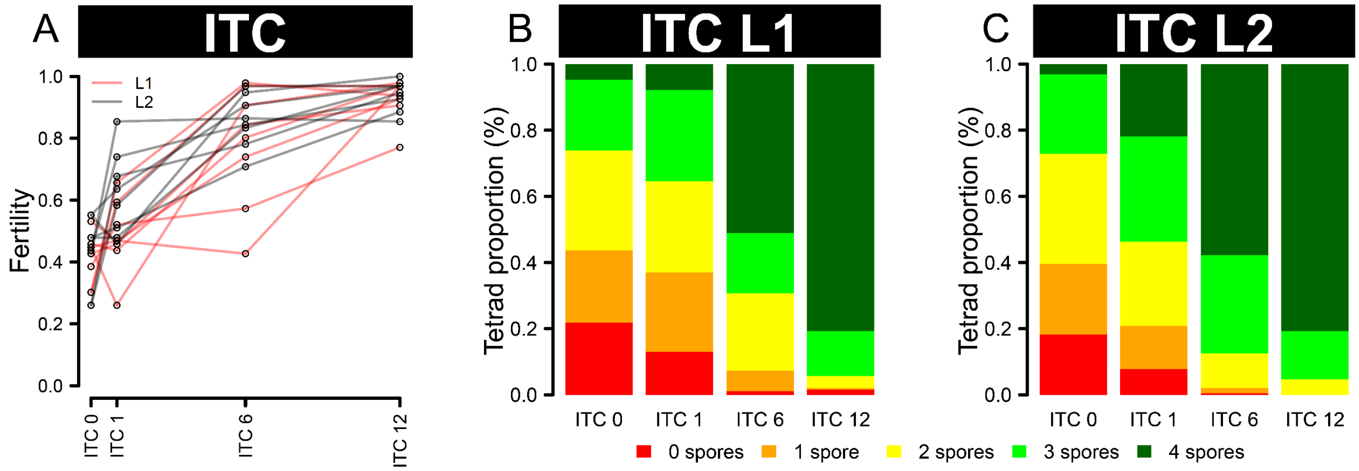

Supplementary Figure 6. Intra-tetrad mating restores hybrid fertility. (**A**) Fertility trajectories of 16 hybrids through 12 sporulations followed by intra-tetrad mating events. Line colors indicate the *SpB* × *SpC* cross identity. (**B**) and (**C**) Combined proportions of tetrad types per cross type after hybridization (ITC 0) and after 1, 6 and 12 intra-tetrad cross rounds. Source data are provided as a Source Data file.

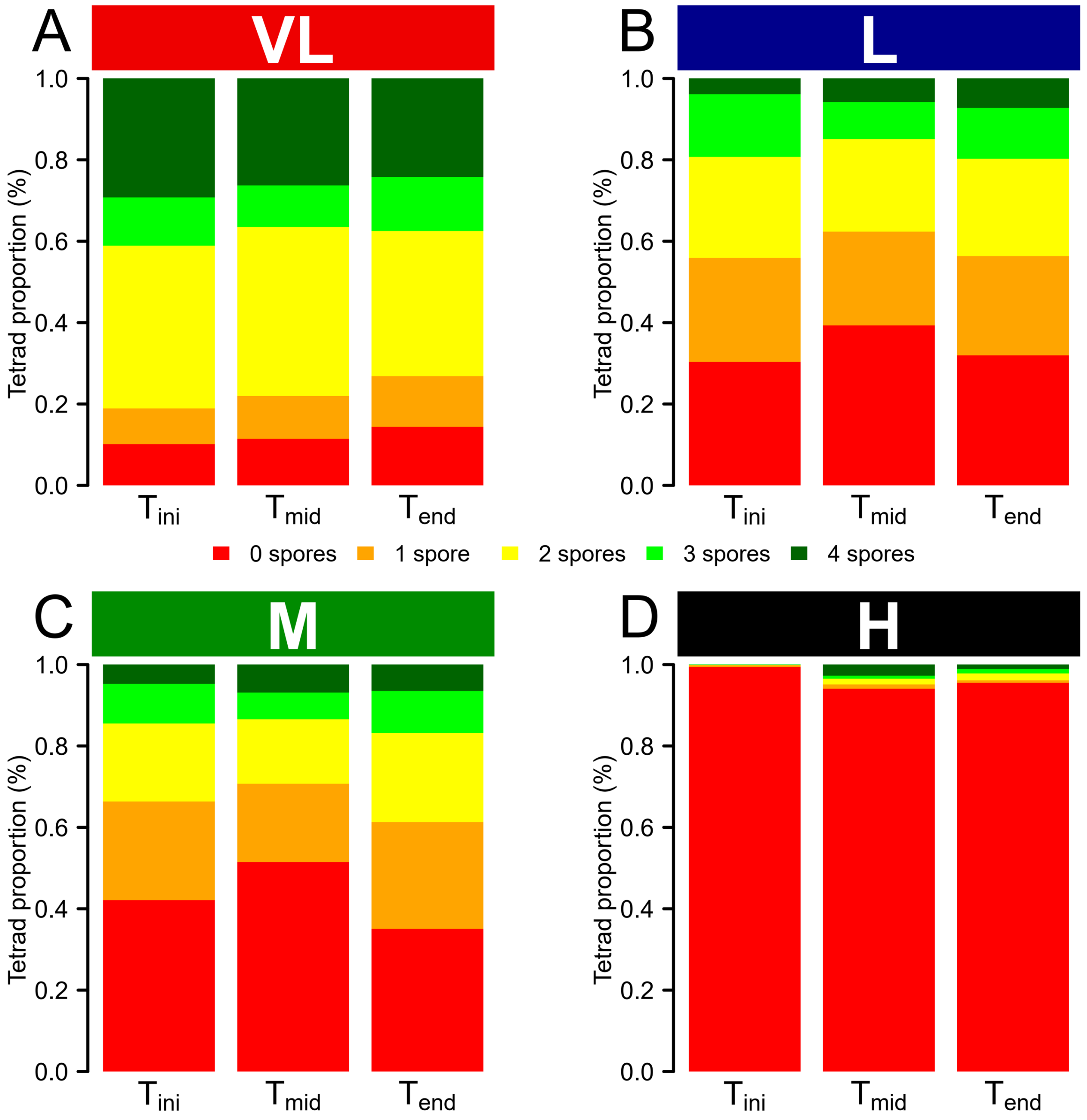

Supplementary Figure 7. The proportions of tetrad types do not change during evolution.

The proportions of possible tetrad types per time point tested for the (**A**) VL_{div}, (**B**) L_{div}, (**C**) M_{div} and (**D**) H_{div} crosses. The data of the two independent crosses for each cross type were merged for this analysis. Source data are provided as a Source Data file.

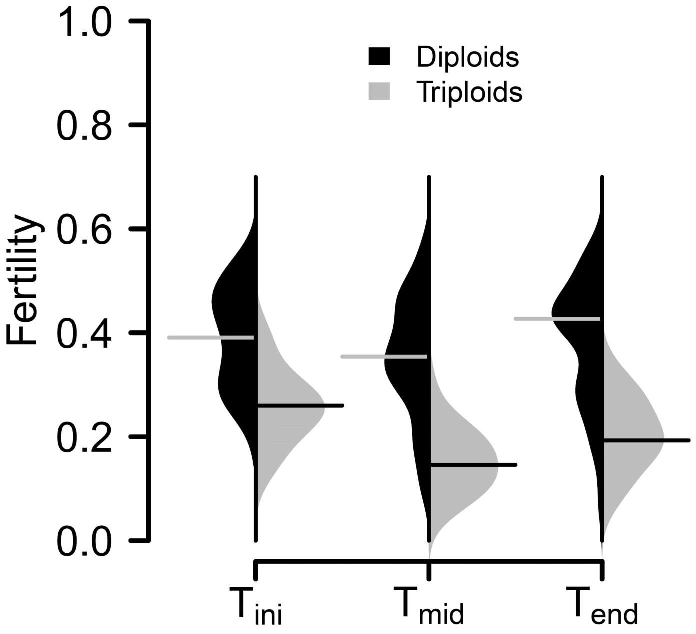

Supplementary Figure 8. Triploid hybrids have a lowered fertility. Distributions of fertility values (fraction of viable spores) for the diploid (black) and triploid (grey) individuals from the L lines at each of the three tested timepoints. The bold lines represent median values. Source data are provided as a Source Data file.

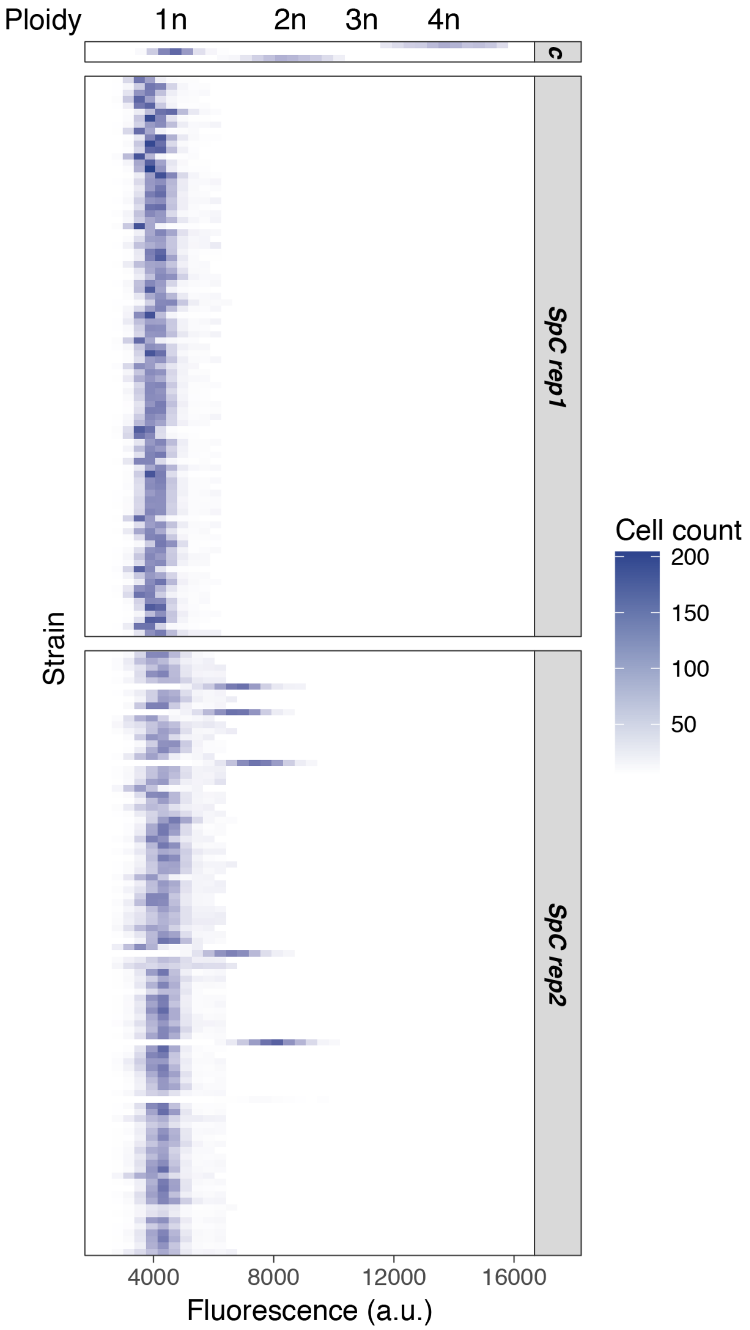

Supplementary Figure 9. The parental *SpC* haploid stocks contain a small fraction of diploids. Ploidy of 94 isolated colonies from the parental *SpC* haploid stocks (LL2011_004 and LL2011_009) using flow cytometry repeated at two independent times. The c panel at the top indicates controls. Source data are provided as a Source Data file.

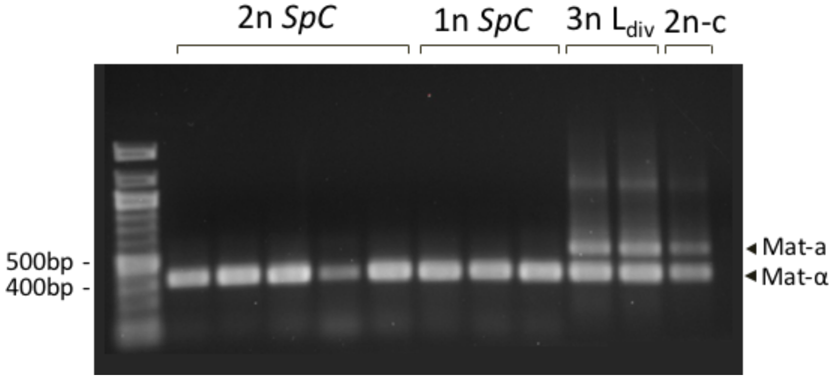

Supplementary Figure 10. The small fraction of diploids in the *SpC* haploid stocks are pseudo-haploids. PCR of the mating type locus on genomic DNA of isolated colonies. The 5 diploids (2n *SpC*) identified in the *SpC* haploid stocks are shown along with 3 haploid *SpC* (1n *SpC*) from the same stocks, two triploid L_{div} lines (3n L_{div}) and a diploid control (2n-c)

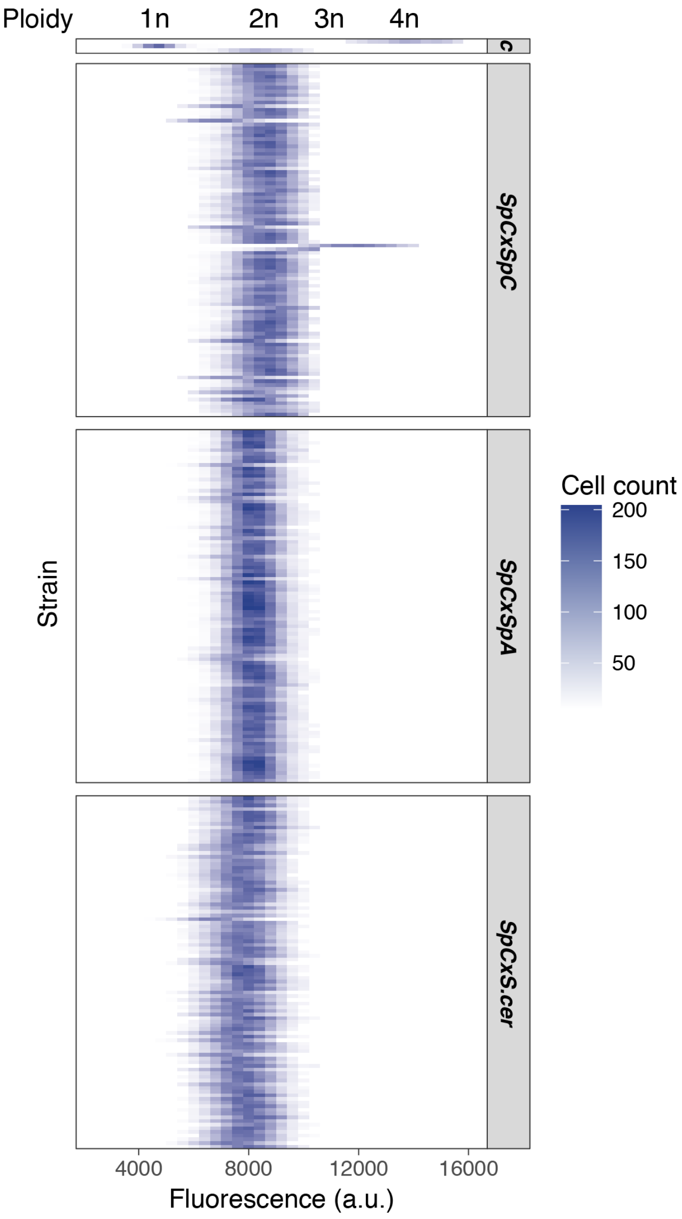

Supplementary Figure 11. Triploidy is rare in hybrids from crosses between *SpC* and other lineages and species. Ploidy of 94 independent replicates of (*SpC* × *SpC*), (*SpC* × *SpA*) and (*SpC* × *S. cerevisiae*) crosses using flow cytometry. The c panel corresponds to controls.

Source data are provided as a Source Data file.

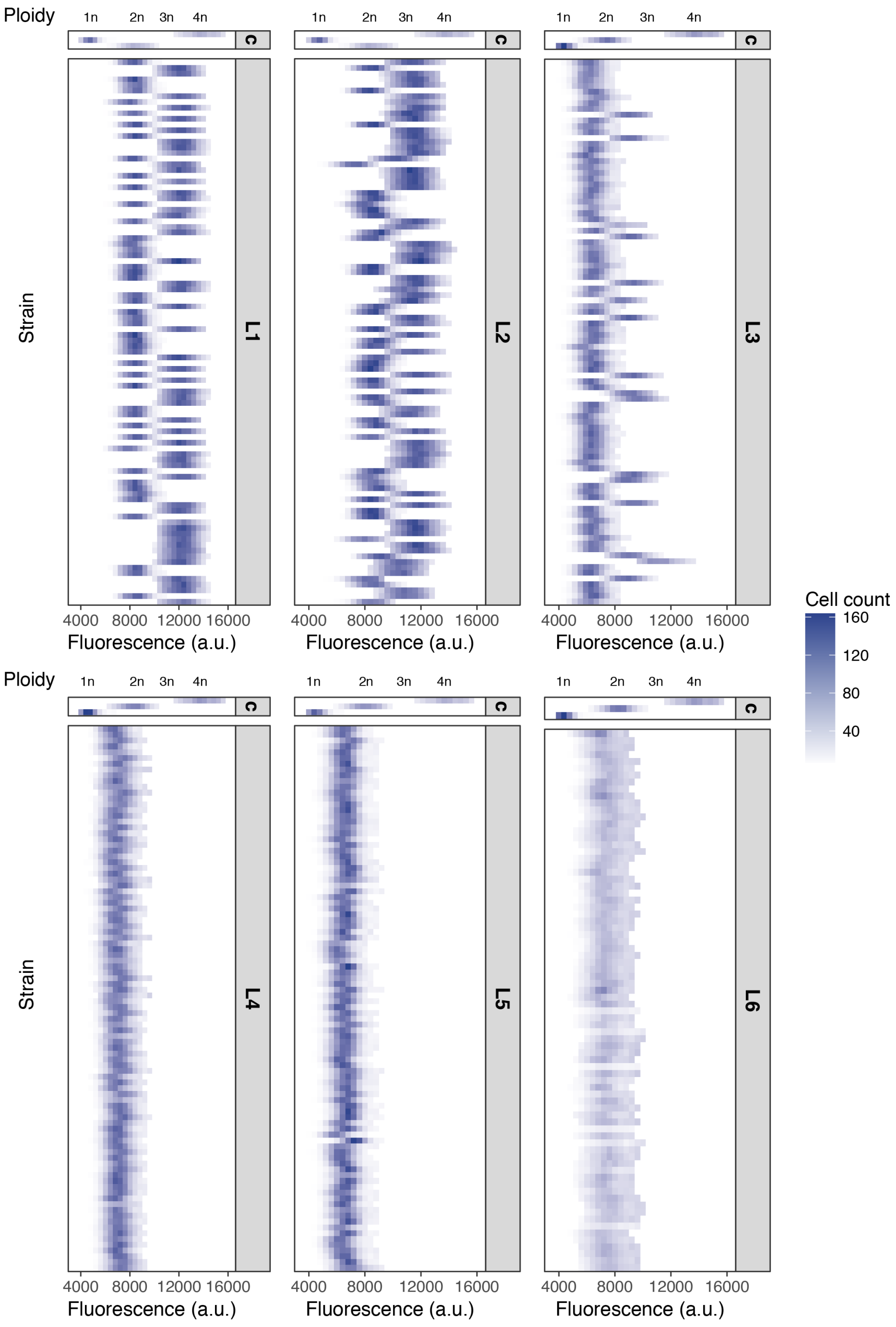

Supplementary Figure 12. Triploidy is observed in crosses between different *SpB* and *SpC* strains but not in all crosses. Ploidy of 94 independent replicates of different *SpB* × *SpC* crosses ($L_{div}1$, $L_{div}2$, $L_{div}3$, $L_{div}4$, $L_{div}5$ and $L_{div}6$). The c panel corresponds to controls. The strains used for each cross are listed in the Supplementary Table 1 and 3. Source data are provided as a Source Data file.

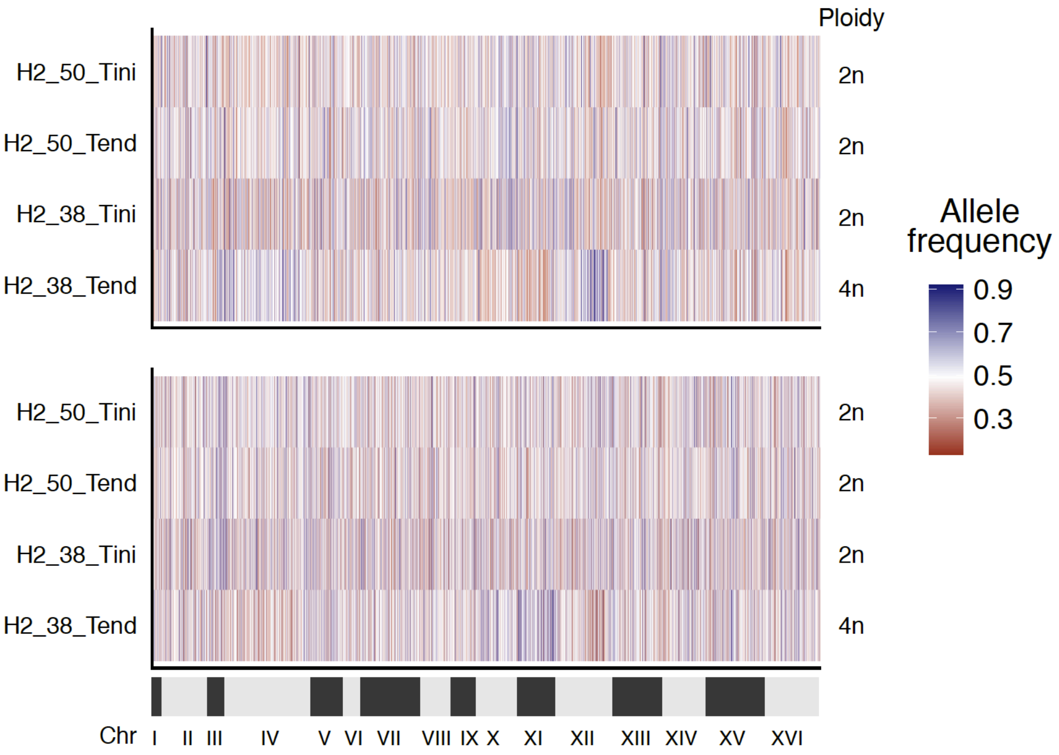

Supplementary Figure 13. The tetraploid hybrids between *S. paradoxus* and *S. cerevisiae* result from a whole genome duplication of both parental genomes. Allele frequencies along the 16 chromosomes of the tetraploid H2_38 at T_{end} are around 50%, similar to what is seen for the H2_38 at T_{ini} and the diploid hybrid H2_50 at T_{ini} and T_{end}. The heatmap on the top represents the allele frequency of reads generated by GBS after mapping on *S. cerevisiae* reference genome (YPS128) (53078 markers) and the one on the bottom after mapping on *S. paradoxus* reference genome (CBS432) (56280 markers), showing consistent results independently from the reference genome used. Source data are provided as a Source Data file.

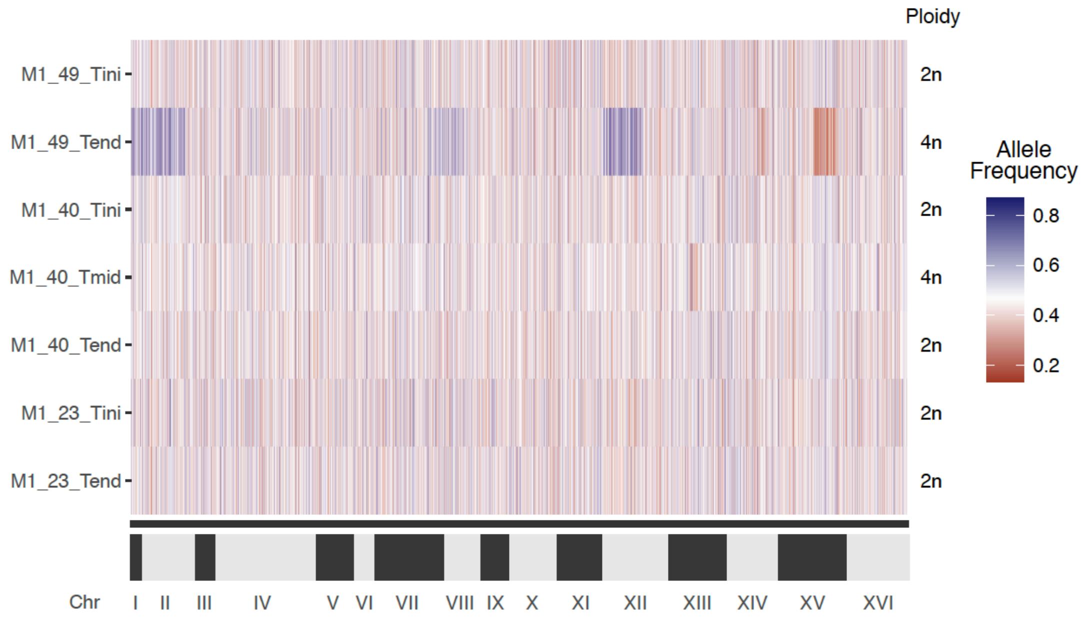

Supplementary Figure 14. The tetraploid hybrids between *SpB* and *SpA* result from a whole genome duplication of both parental genomes.

Allele frequencies along the 16 chromosomes of the tetraploid M1_40 at T_{mid} and M1_49 at T_{end} are around 50%, similar to what is seen for the M1_49 at T_{ini} and M1_40 at T_{ini} and T_{end} and the diploid hybrid M1_23 at T_{ini} and T_{end}. The heatmap represents allele frequencies after mapping reads generated by GBS on the *S. paradoxus* reference genome (CBS432) (16020 markers). Source data are provided as a Source Data file.

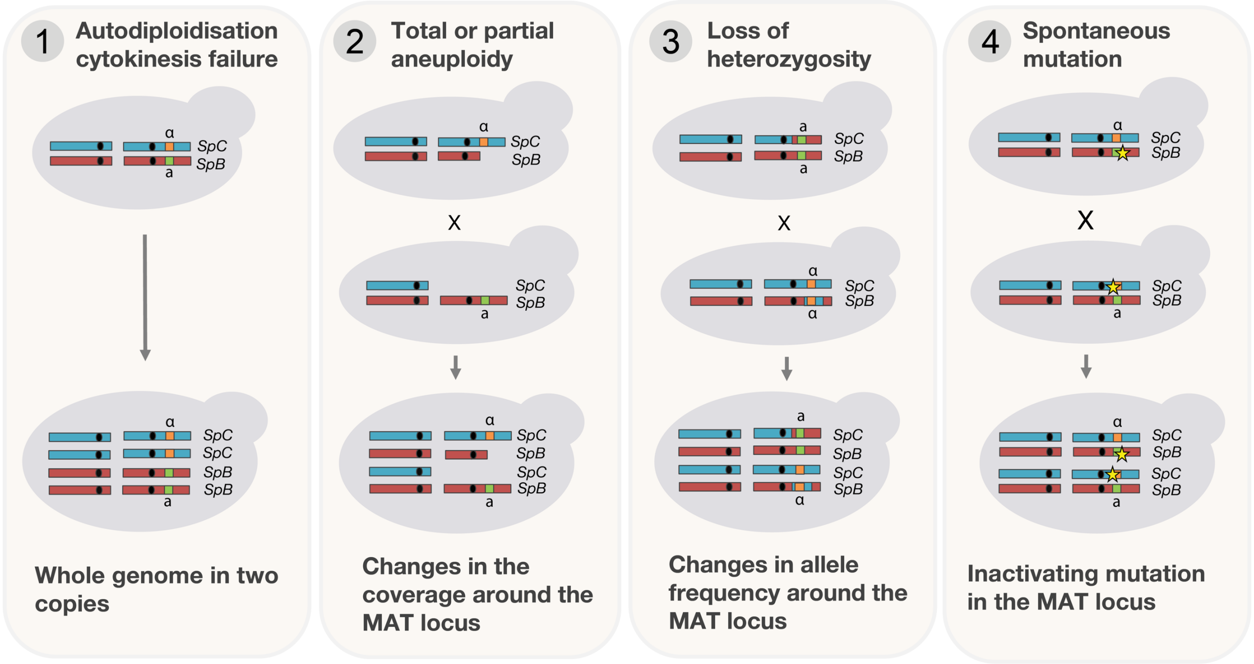

Supplementary Figure 15. Major potential mechanisms of whole-genome doubling

Whole genome doubling could occur by (1) autodiploidization, which is a consequence of cytokinesis failure. Whole genome doubling could also be caused by means of damage to one copy of the *MAT* locus. Damage to the *MAT* locus could be caused by (2) partial or complete chromosome loss of the chromosome containing the *MAT* locus (chromosome III), (3) loss of heterozygosity around the *MAT* locus, (4) or an inactivating mutation in one copy of the *MAT* locus.

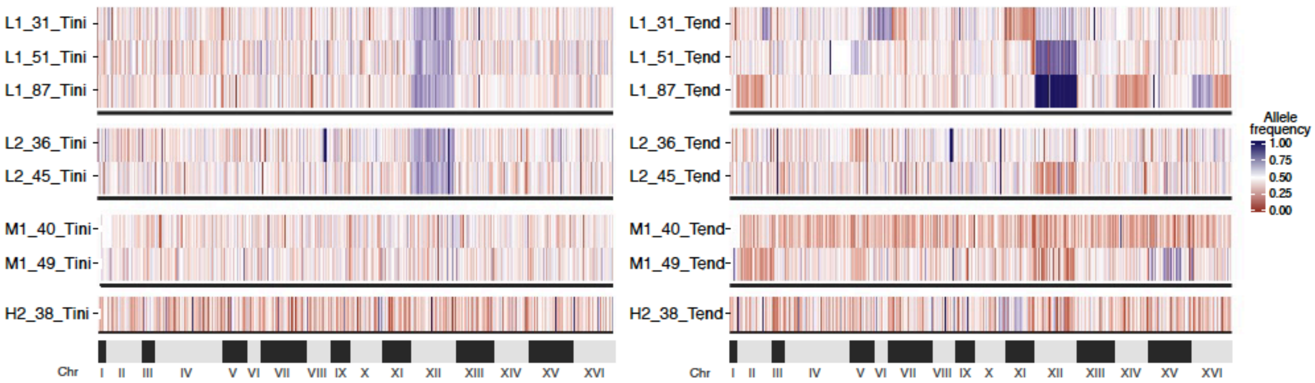

Supplementary Figure 16. The tetraploid hybrids result from a whole genome duplication of both parental genomes. Allele frequencies along the 16 chromosomes of the tetraploid hybrids at T_{end} are around 50%, similar to what is seen for the same diploid hybrids at T_{ini}. The heatmap represents allele frequencies after mapping reads generated by whole genome sequencing on the *S. paradoxus* reference genome (MSH604) (276003 markers for L1 lines, 280756 markers for L2 lines, 471547 markers for M1 lines and 981580 markers for the H2_38).

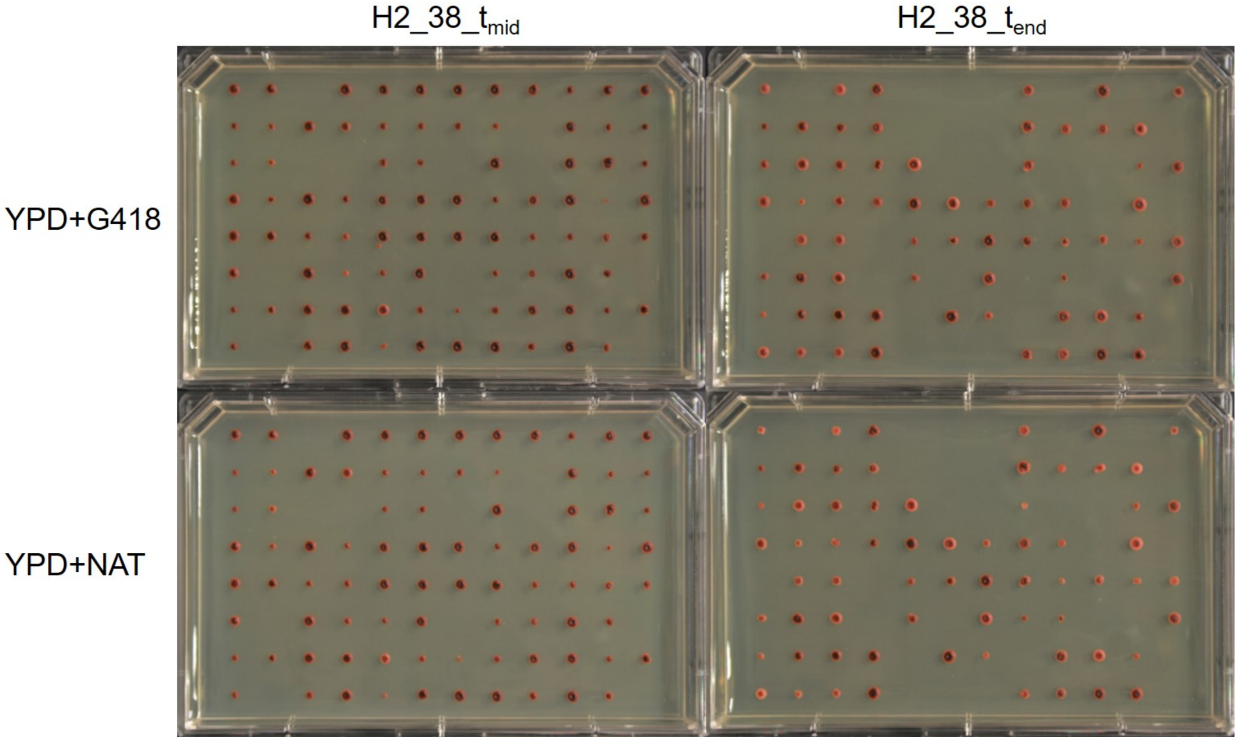

Supplementary Figure 17. The *S. paradoxus* × *S. cerevisiae* (H2) hybrids segregate a copy of each parental genome in its diploid spores. Spores dissected at T_{mid} and T_{end} from the tetraploid line H2_38 were plated on selective media to assess the meiotic segregation of the selection cassettes that were introduced at the *HO* locus. The *S. cerevisiae* genome harbor the NAT resistance while *S. paradoxus* genome harbor the G418 resistance at the *HO* locus (Chr IV). Empty spaces on plates represent spores that did not form colonies on the original dissection plate. As all the viable spores inherited both resistances, it is likely that the spores contain full non-recombined *S. cerevisiae* and *S. paradoxus* haplotypes.

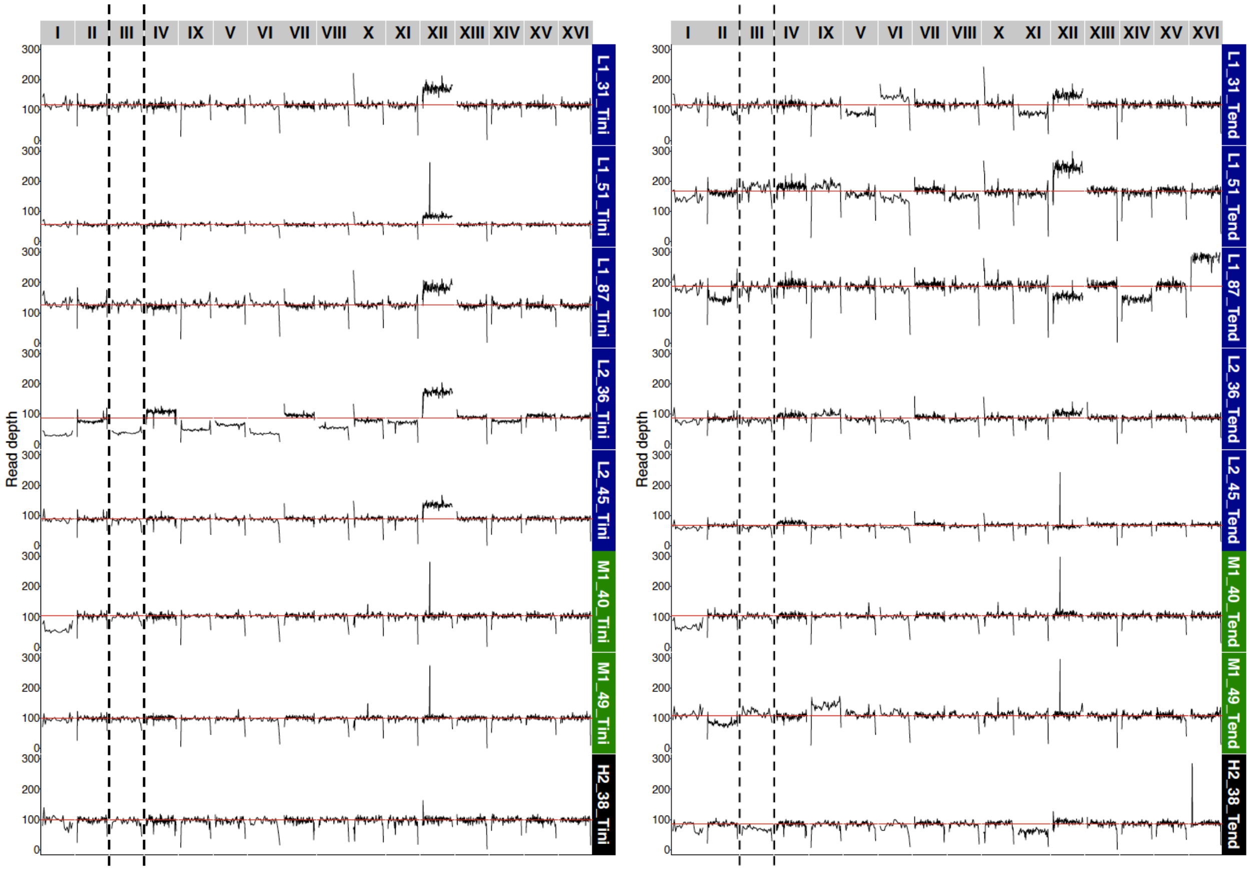

Supplementary Figure 18. The loss of chromosome III is not the molecular mechanism leading to whole genome doubling. Sequencing read depth for bins of 10 kb on the 16 chromosomes of the 8 tetraploid lines at T_{ini} (the left panel) and T_{end} (the right panel). The red line represents the average sequencing read depth of the whole genome. Several aneuploidies are detected in almost all hybrids at T_{ini} and T_{end}. Source data are provided as a Source Data file.

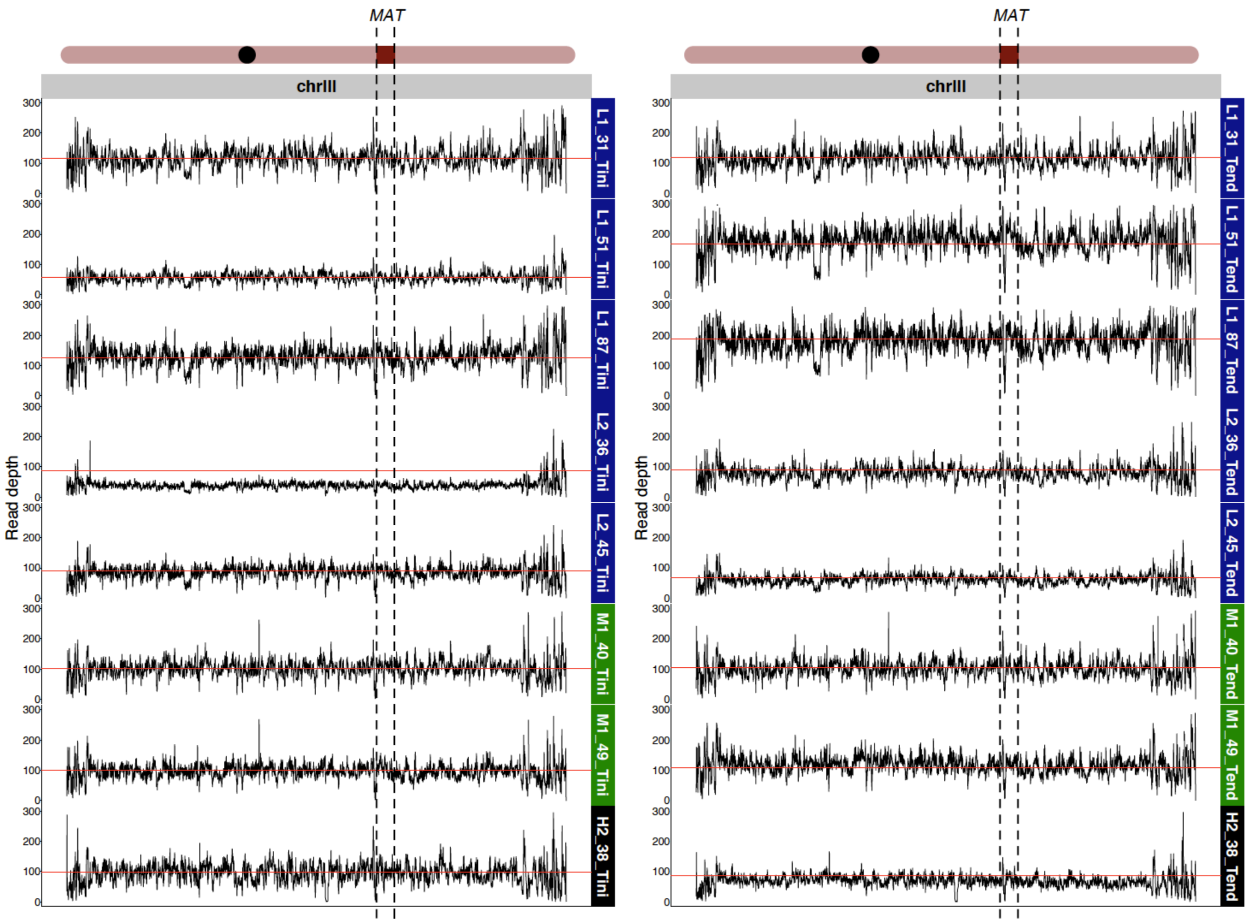

Supplementary Figure 19. The partial loss of Chromosome III is unlikely to be the molecular mechanism leading to whole genome doubling. Sequencing read depth for each bin of 100 bp on chromosome III for the 8 tetraploid lines at T_{ini} (the left panel) and T_{end} (the right panel). The red lines represent the average sequencing read depth of the whole genome. The active mating type locus (*MAT*) is indicated by dashed lines. Source data are provided as a Source Data file.

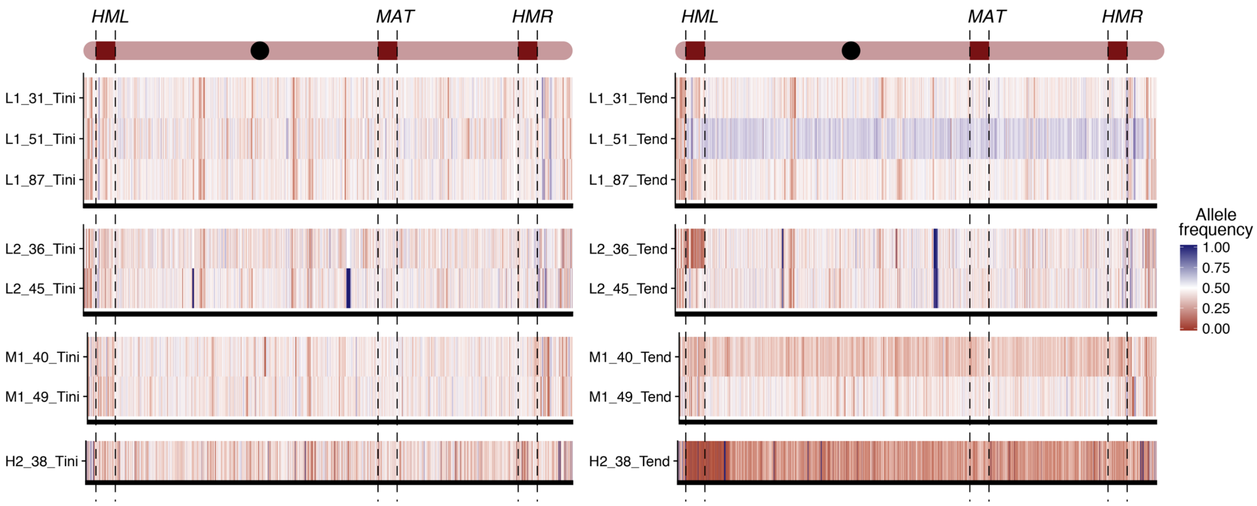

Supplementary Figure 20. Loss of heterozygosity around the *MAT* locus is most likely not the molecular mechanism causing whole genome doubling. Allele frequencies along the chromosome III for the 8 tetraploid lines at T_{ini} (left panel) and T_{end} (right panel). The active mating type locus (*MAT*) and the silent mating type loci (*HML* and *HMR*) are indicated by dashed lines. The heatmaps represent allele frequency after mapping on *S. paradoxus* reference genome (6611 markers for L1 lines, 5986 markers for L2 lines, 11794 markers for M1 lines and 20537 markers for the H2_38). Source data are provided as a Source Data file.

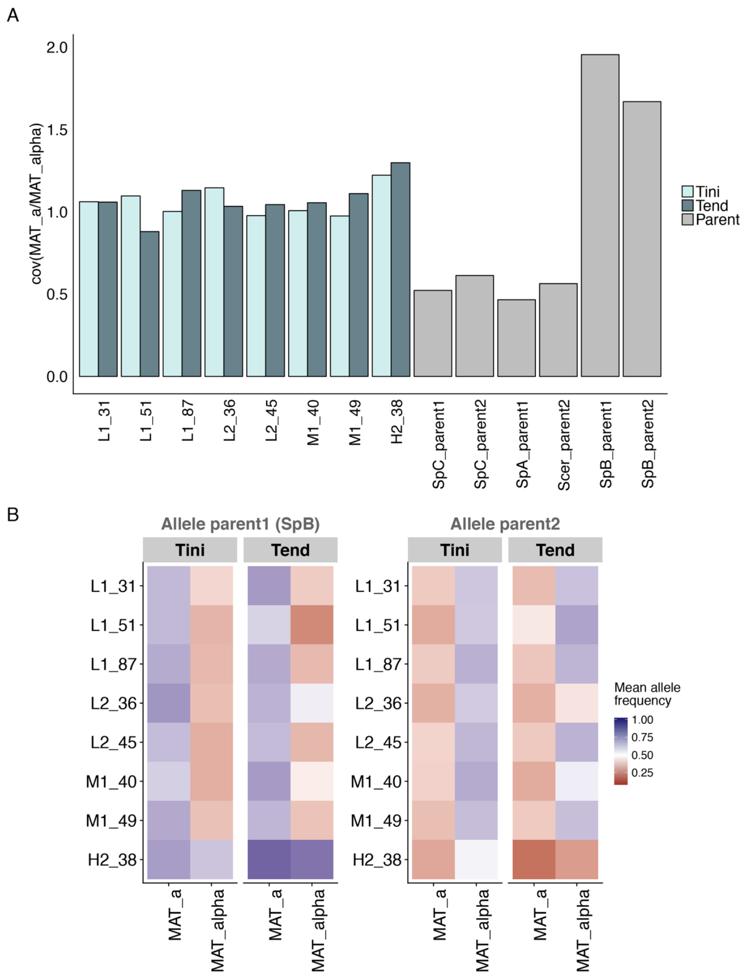

Supplementary Figure 21. Copy number variation and allele frequency of *MAT*a and *MAT*α sequences of tetraploid hybrids show no double damage at the *MAT* locus.

(A) The bar plots represent the ratio of *MAT*a and *MAT*α average read depth for the 8 tetraploid lines at T_{ini} and T_{end} as well as the 6 haploid parental strains. The average sequencing read depth of *MAT*a and *MAT*α corresponding sequences were calculated for a sequence of 1.3 kb containing SNPs that differentiates the two copies. (B) The heatmaps show the average allele frequencies (AF) of the *MAT*a and *MAT*α sequences for the 8 tetraploid lines at T_{ini} and T_{end}. After mapping reads on *S. paradoxus MAT*a and *MAT*α reference sequences, the average AF were calculated for a sequence of 1.3 kb containing SNPs that differentiate the two mating type copies. The panel on the left shows the average allele frequencies of both mating type sequences corresponding to the haploid *SpB* parent (which is *MAT*a) alleles and the panel on the right those corresponding to the haploid *SpC* (for L1 and L2 lines), *SpA* (for M1 lines) or *S. cerevisiae* (for H2_38) parents (which are *MAT*α) alleles. Source data are provided as a Source Data file.

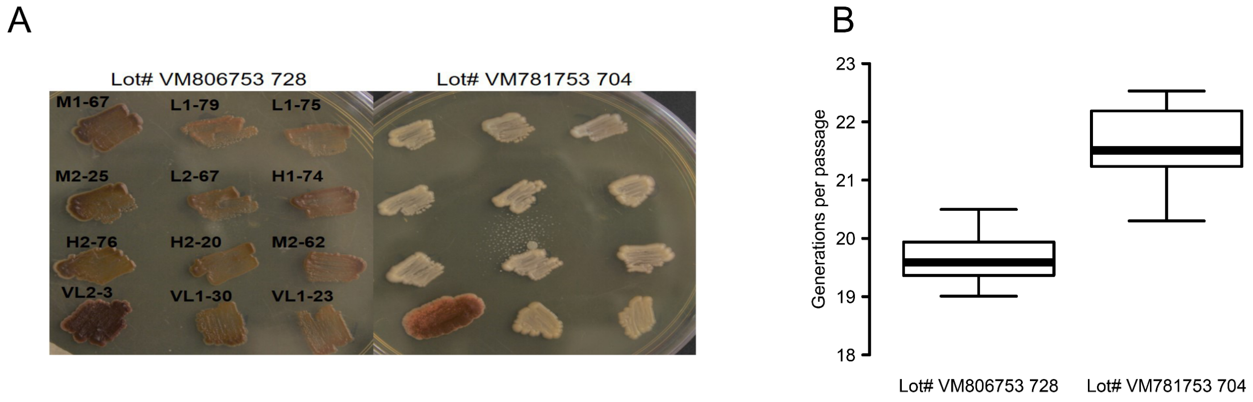

Supplementary Figure 22. The *ade2Δ* coloration phenotype and growth rate is media dependent. (**A**) Sporulation negative strains appear to be red when grown on YPD prepared with yeast extract from one lot (panel A left). Using the same ingredients but changing the lot number of the yeast extract yielded a very different coloration for all tested lines but one (panel A right). Evolution line numbers indicated above the cell patches. Plates were prepared and inoculated on the same day. Photos were taken after 5 days of growth. (**B**) Generations per passage on the two different media measured by cytometry on a subset of triploids and tetraploids that were sporulation positive. For all boxplots the bold center line corresponds to the median value, the box boundaries correspond to the 25th and the 75th percentile, the whiskers correspond to 1.5 times the inter-quartile range and the dots to outlier values. Source data are provided as a Source Data file.

Supplementary Table 1.
List of strains used in this study

Strain	Sampling site	Lineage	Mating type	Genotype	Reference Wild	Reference hoΔ	Reference ade2Δ
YPS644	Buck Hill Falls, PE	*SpA*	α	hoΔ::KAN, ade2Δ::HPH	Kuehne *et al*. 2007	Charron *et al*. 2014	This study
YPS644	Buck Hill Falls, PE	*SpA*	a	hoΔ::KAN, ade2Δ::HPH	Kuehne *et al*. 2007	Charron *et al*. 2014	This study
YPS744	Tuscarora Forest, PE	*SpA*	α	hoΔ::NAT, ade2Δ::HPH	Kuehne *et al*. 2007	Charron *et al*. 2014	This study
YPS744	Tuscarora Forest, PE	*SpA*	a	hoΔ::NAT, ade2Δ::HPH	Kuehne *et al*. 2007	Charron *et al*. 2014	This study
UWOPS_91_202	Long Point, ON	*SpB*	a	hoΔ::KAN, ade2Δ::HPH	Kuehne *et al*. 2007	Charron *et al*. 2014	This study
UWOPS_91_202	Long Point, ON	*SpB*	a	hoΔ::NAT	Kuehne *et al*. 2007	Charron *et al*. 2014	-
MSH604	Mont-St-Hilaire, QC	*SpB*	a	hoΔ::NAT, ade2Δ::HPH	Leducq *et al*. 2014	Leducq *et al*. 2016	This study
LL12_021	Pointe Platon, QC	*SpB*	α	hoΔ::NAT, ade2Δ::HPH	Leducq *et al*. 2014	Leducq *et al*. 2016	This study
LL12_028	Sherbrooke, QC	*SpB*	α	hoΔ::KAN, ade2Δ::HPH	Leducq *et al*. 2014	Leducq *et al*. 2016	This study
YPS484	Grand Bend, ON	*SpB*	a	hoΔ::HPH	Kuehne et al. 2007	Charron *et al*. 2014	-
yHKS226	Plainfield, NH	*SpB*	α	hoΔ::HPH	Sylvester *et al*. 2015	Leducq *et al*. 2016	-
LL11_004	Cap Chat, QC	*SpC*	α	hoΔ::KAN, ade2Δ::HPH	Leducq *et al*. 2014	Charron *et al*. 2014	This study
LL11_009	St-Michel-du-Squatec, QC	*SpC*	α	hoΔ::NAT, ade2Δ::HPH	Leducq *et al*. 2014	Charron *et al*. 2014	This study
LL11_009	St-Michel-du-Squatec, QC	*SpC*	a	hoΔ::NAT, ade2Δ::HPH	Leducq *et al*. 2014	Charron *et al*. 2014	This study
MSH_587-1	Mont-St-Hilaire, QC	SpC	α	hoΔ::HPH	Leducq et al. 2014	Charron et al. 2014	-
YPS667	Buck Hill Falls, PE	SpC	α	hoΔ::HPH	Kuehne et al. 2007	Charron et al. 2014	-
yHKS225	Plainfield, NH	SpC	a	hoΔ::NAT	Sylvester et al. 2015	Leducq et al. 2016	-
LL12_019	Pointe Platon, QC	SpC	a	hoΔ::HPH	Leducq et al. 2014	Leducq et al. 2016	-
LL13_040	Rockport, MA	*Scer*	α	hoΔ::KAN, ade2Δ::HPH	Leducq et al. 2016	This study	This study
LL13_040	Rockport, MA	*Scer*	a	hoΔ::KAN, ade2Δ::HPH	Leducq et al. 2016	This study	This study
LL13_054	Rockport, MA	*Scer*	α	hoΔ::NAT, ade2Δ::HPH	Leducq et al. 2016	This study	This study
LL13_054	Rockport, MA	*Scer*	a	hoΔ::NAT, ade2Δ::HPH	Leducq et al. 2016	This study	This study

Supplementary Table 2.

Genetic divergence among the different *S. paradoxus* lineages and among different strains of the same lineage[8].

Reference	Compared to	% Mean nucleotide divergence
SpB	*SpB*	0.424
SpB	*SpC*	2.237
SpB	*SpA*	3.748
SpC	*SpC*	0.288
SpC	*SpA*	3.432
SpA	*SpA*	0.101

Supplementary Table 3.
List of crosses performed for this study

Cross	a strain	a strain resistance	α strain	α strain resistance
$VL_{div}1$	MSH604	NAT	LL12_028	G418
$L_{div}1$	MSH604	NAT	LL11_004	G418
$M_{div}1$	MSH604	NAT	YPS644	G418
$H_{div}1$	MSH604	NAT	LL13_040	G418
$VL_{div}2$	UWOPS_91_202	G418	LL12_021	NAT
$L_{div}2$	UWOPS_91_202	G418	LL11_009	NAT
$M_{div}2$	UWOPS_91_202	G418	YPS744	NAT
$H_{div}2$	UWOPS_91_202	G418	LL13_054	NAT
$L_{div}3$	UWOPS_91_202	NAT	MSH587	HYG
$L_{div}4$	YPS484	NAT	YPS667	HYG
$L_{div}5$	yHKS226	HYG	yHKS225	NAT
$L_{div}6$	LL12_021	NAT	LL12_019	HYG
SpC×*SpA*-1	YPS744	NAT	LL11_004	G418
SpC×*SpA*-2	YPS644	G418	LL11_009	NAT
SpC×*SpC*-1	LL11_009	NAT	LL11_004	G418
SpC×*Scer*-1	LL13_054	NAT	LL11_004	G418
SpC×*Scer*-2	LL13_040	G418	LL11_009	NAT

Supplementary Table 4.
Logrank test P-values for all survival curves

Cross type

	M	VL	L
VL	0.010114	NA	NA
L	0.000305	2.49E-06	NA
H	0.000786	3.45E-06	0.667319

Blocks

	set1(1-64)	set1(65-96)	set2(1-64)	set2(65-96)	VL1
set1(65-96)	0.075385	NA	NA	NA	NA
set2(1-64)	0.018042	0.907645	NA	NA	NA
set2(65-96)	0.147398	0.768516	0.689276	NA	NA
VL1	0.003756	0.042042	0.042042	0.025266	NA
VL2	0.00077	0.009335	0.009335	0.006103	0.388572

Groups

	LH
VLM	5.22E-09

Supplementary Table 5.
Number of generations equivalence for the different time points sampled

Last Glycerol	T_{ini} (generation number)	T_{mid} (generation number)	T_{end} (generation number)
P4	P0	NA	P4 (88)
P7	P0	P4 (88)	P7 (176)
P10	P0	P4 (88)	P10 (242)
P13	P0	P7 (176)	P13 (308)
P16	P0	P7 (176)	P16 (352)
P19	P0	P10 (242)	P19 (440)
P22	P0	P10 (242)	P22 (506)
P25	P0	P13 (308)	P25 (572)
P28	P0	P13 (308)	P28 (638)
P31	P0	P16 (352)	P31 (704)
P35	P0	P16 (352)	P35 (770)

Supplementary Table 6

P-values of the Fisher's exact tests on the proportions of viable spores for the 23 lines that were significantly different between T_{ini} and T_{end} (correction with FDR)

Lines	T_{ini}-T_{mid}	T_{ini}-T_{mid}_corrected	T_{mid}-T_{end}	T_{mid}-T_{end}_corrected	T_{ini}-T_{end}	T_{ini}-T_{end}_corrected
L1_30	0.0002	0.0033	0.4702	0.8408	0.0047	0.0360
L1_31	0.0371	0.1720	0.3865	0.7330	0.0020	0.0189
L1_41	0.1155	0.3965	0.0736	0.2872	0.0004	0.0055
L1_51	1.28E-11	4.40E-10	0.1975	0.5164	5.68E-16	2.92E-14
L1_63	0.1275	0.4184	0.2223	0.5612	0.0038	0.0304
L1_87	0.0699	0.2789	7.25E-17	4.67E-15	6.99E-11	2.25E-09
L2_10	0.0032	0.0266	1	1	0.0032	0.0266
L2_22	0.0107	0.0693	0.3914	0.7330	0.0003	0.0044
L2_36	1.71E-23	1.76E-21	0.4973	0.8655	9.48E-27	1.22E-24
L2_42	0.0195	0.1128	0.3480	0.6920	0.0006	0.0074
L2_45	9.82E-11	2.97E-09	0.4975	0.8655	1.54E-08	4.17E-07
L2_89	0.0006	0.0071	1	1	2.60E-04	0.004
M1_43	0.0371	0.1720	0.0087	0.0584	1.48E-06	3.30E-05
M1_49	3.34E-13	1.32E-11	0.0008	0.0086	7.66E-05	0.0013
M1_75	2.55E-07	5.97E-06	1	1	2.55E-07	5.97E-06
M2_86	0.5711	0.9278	9.90E-04	0.0102	5.83E-05	0.0010
M2_95	0.0561	0.2387	1.46E-03	0.0142	2.13E-07	5.49E-06
H2_38	7.46E-48	3.84E-45	2.27E-05	0.0004	1.19E-28	3.06E-26
VL1_1	0.8852	1	0.0126	0.0773	0.0054	0.0411
VL1_43	0.0031	0.0266	0.3206	0.6527	4.39E-05	7.80E-04
VL1_45	4.21E-06	9.04E-05	0.0304	0.1468	1.19E-11	4.37E-10
VL2_6	0.0973	0.3631	1.41E-14	6.07E-13	1.01E-20	7.40E-19
VL2_25	1	1	5.60E-16	2.92E-14	1.85E-15	8.67E-14

Supplementary Table 7.
Ploidy distribution of 94 independent hybrids in three replicates of the $L_{div}1$ and $L_{div}2$ crosses.

	Ploidy		
Cross	2n (%)	3n (%)	4n (%)
$L_{div}1$-1[*]	48.0	52.0	0.0
$L_{div}1$-2	98.0	2.0	0.0
$L_{div}1$-3	67.0	32.0	1.0
$L_{div}2$-1[*]	42.5	57.5	0.0
$L_{div}2$-2	91.5	8.5	0.0
$L_{div}2$-3	99.0	1.0	0.0

[*]crosses used for the evolution experiment

Supplementary Table 8.
Oligonucleotides used in this study

Primer name	Sequence	Description
CLOP97-F1	acaatcaaggaatcaagaaaccgtggtaaaaaattcaagtCAGCTGAAGCTTCGTACGC	Forward primer to construct the deletion cassette for the *ADE2* gene in *SpA*
CLOP97-F2	gtaattgttcgctggccaagtatatcaatacatttatataGCATAGGCCACTAGTGGATC	Reverse primer to construct the deletion cassette for the *ADE2* gene in *SpA*
CLOP97-F3	acaatcaaggaatcaagaaaccgtgataaaaaattcaagtCAGCTGAAGCTTCGTACGC	Forward primer to construct the deletion cassette for the *ADE2* gene in *SpB*
CLOP97-F4	tgtgattgttgctggccaagtacatcaatacatttatacaGCATAGGCCACTAGTGGATC	Reverse primer to construct the deletion cassette for the *ADE2* gene in *SpB*
CLOP97-F5	acaattaaggaatcaagaaaccgtgataaaaaattcaagtCAGCTGAAGCTTCGTACGC	Forward primer to construct the deletion cassette for the *ADE2* gene in *SpC*
CLOP97-F6	gtaattgttcgctggccaagtatattaatacatttatataGCATAGGCCACTAGTGGATC	Reverse primer to construct the deletion cassette for the *ADE2* gene in *SpC*
CLOP97-F7	caatcaagaaaaacaagaaaatcggacaaaacaatcaagtCAGCTGAAGCTTCGTACGC	Forward primer to construct the deletion cassette for the *ADE2* gene in *Scer*
CLOP97-F8	ataattatttgctgtgcaagtatatcaataaacttatataGCATAGGCCACTAGTGGATC	Reverse primer to construct the deletion cassette for the *ADE2* gene in *Scer*
CLOP97-F9	GGACCAGATGGACGATATTA	Forward primer for the verification of the *ADE2* gene deletion in *S. paradoxus*
CLOP97-F10	CGGTAATGCATTGAGCAGAT	Reverse primer for the verification of the *ADE2* gene deletion in *S. paradoxus*
CLOP97-F11	GACTCTTGTTGCATGGCTAC	Forward primer for the verification of the *ADE2* gene deletion in *S. cerevisiae*
CLOP97-F12	CGCAGACTTAAGCAGGTAAT	Reverse primer for the verification of the *ADE2* gene deletion in *S. cerevisiae*
verifMATa_F	AGTCACATCAAGATCGTTTATGG	Forward primer for mating type diagnostic (a specific)
verifMATalpha_F	ACTCCACTTCAAGTAAGAGTTTG	Forward primer for mating type diagnostic (alpha specific)
verifMATa/alpha_R	GCACGGAATATGGGACTACTTCG	Reverse primer for mating type diagnostic
CLOP80-A3	TGAGGTCCCGCATGAATGAC	Forward primer for rnl diagnostic
CLOP80-B3	ACGTACTTGTTTCACTCGTTTGT	Reverse primer for rnl diagnostic
CLOP80-C3	GGTTCAAGATGATTAATTTCACAAG	Forward primer for atp6 diagnostic
CLOP80-D3	ACCAGCAGGTACGAATAATGA	Reverse primer for atp6 diagnostic

Supplementary references:

1 Klar, A. J., Hicks, J. B. & Strathern, J. N. Directionality of yeast mating-type interconversion. *Cell* **28**, 551-561 (1982).

2 Leducq, J. B. *et al.* Speciation driven by hybridization and chromosomal plasticity in a wild yeast. *Nat Microbiol* **1**, 15003, doi:10.1038/nmicrobiol.2015.3 (2016).

3 Yue, J.-X. *et al.* Contrasting evolutionary genome dynamics between domesticated and wild yeasts. *Nature Genetics* **49**, 913, doi:10.1038/ng.3847 (2017).

4 Kuehne, H. A., Murphy, H. A., Francis, C. A. & Sniegowski, P. D. Allopatric divergence, secondary contact, and genetic isolation in wild yeast populations. *Curr Biol* **17**, 407-411, doi:10.1016/j.cub.2006.12.047 (2007).

5 Charron, G., Leducq, J. B. & Landry, C. R. Chromosomal variation segregates within incipient species and correlates with reproductive isolation. *Mol Ecol* **23**, 4362-4372, doi:10.1111/mec.12864 (2014).

6 Leducq, J. B. *et al.* Local climatic adaptation in a widespread microorganism. *Proc Biol Sci* **281**, 20132472, doi:10.1098/rspb.2013.2472 (2014).

7 Sylvester, K. *et al.* Temperature and host preferences drive the diversification of Saccharomyces and other yeasts: a survey and the discovery of eight new yeast species. *FEMS Yeast Res* **15**, doi:10.1093/femsyr/fov002 (2015).

8 Eberlein, C. *et al.* Hybridization is a recurrent evolutionary stimulus in wild yeast speciation. *Nat Commun* **10**, 923, doi:10.1038/s41467-019-08809-7 (2019).
