# Supplementary material for: Spontaneous whole-genome duplication restores fertility in interspecific hybrids
Source: Nat Commun. 2019 Sep 11;10:4126. doi: 10.1038/s41467-019-12041-8 (PMC6739354; doi:10.1038/s41467-019-12041-8)
Supplement: Supplementary file 4 — Description of Additional Supplementary Files [file 41467_2019_12041_MOESM4_ESM.pdf]

Description of Additional Supplementary Files

File Name: Supplementary Data 1
Description: Zip file containing data for fertility (evolution, ITC and autodiploidization), generation number per passage, survival of the strains and the custom R script for the analyses and to generate Figures. Also contains the custom R scripts for the analyses of ploidy, GBS and Illumina sequencing data.

File Name: Supplementary Data 2
Description: Zip file containing data from mtDNA genotyping, colony coloration analysis and growth measurements on glycerol. The custom Python scripts used for the analysis and figures are provided.
