# Supplementary material for: Spontaneous whole-genome duplication restores fertility in interspecific hybrids
Source: Nat Commun. 2019 Sep 11;10:4126. doi: 10.1038/s41467-019-12041-8 (PMC6739354; doi:10.1038/s41467-019-12041-8)
Supplement: Supplementary file 6 — Supplementary Data 2 [file 41467_2019_12041_MOESM6_ESM.zip › mito_table.pdf]

L1	A75	T_{ini}	+
H1	C74	T_{ini}	+
L1	A79	T_{ini}	+
H1	C83	T_{ini}	+
M1	B67	T_{ini}	+
L2	D67	T_{ini}	+
H1	C24	T_{ini}	+
M2	E23	T_{ini}	+
H1	C31	T_{ini}	+
M2	E25	T_{ini}	+
H1	C34	T_{ini}	+
M2	E34	T_{ini}	+
H1	C38	T_{ini}	+
M2	E62	T_{ini}	+
H1	C51	T_{ini}	-
M2	E65	T_{ini}	+
M2	E70	T_{ini}	+
H2	F69	T_{ini}	+
M2	E81	T_{ini}	+
H2	F76	T_{ini}	+
H2	F12	T_{ini}	+
H2	F8	T_{ini}	+
H2	F15	T_{ini}	+
H2	F80	T_{ini}	+
H2	F20	T_{ini}	+
H2	F90	T_{ini}	+
H2	F21	T_{ini}	+
VL2	H23	T_{ini}	+
H2	F48	T_{ini}	+
VL2	H30	T_{ini}	+
H2	F65	T_{ini}	+
VL1	I3	T_{ini}	+

L1	A36	T_{ini}	+
L1	A75	T_{end}	-
M1	B24	T_{ini}	+
L1	A79	T_{end}	-
L2	D10	T_{ini}	+
M1	B67	T_{end}	-
M2	E42	T_{ini}	+
H1	C24	T_{end}	-
H1	C10	T_{ini}	+
H1	C31	T_{end}	-
H2	F23	T_{ini}	+
H1	C34	T_{end}	-
VL2	H3	T_{ini}	+
H1	C38	T_{end}	-
VL1	I15	T_{ini}	+
H1	C51	T_{end}	-
H1	C74	T_{end}	-
M2	E70	T_{end}	-
H1	C83	T_{end}	-
M2	E81	T_{end}	-
L2	D67	T_{end}	-
H2	F12	T_{end}	-
M2	E23	T_{end}	-
H2	F15	T_{end}	-
M2	E25	T_{end}	-
H2	F20	T_{end}	-
M2	E34	T_{end}	-
H2	F21	T_{end}	-
M2	E62	T_{end}	-
H2	F48	T_{end}	-
M2	E65	T_{end}	-
H2	F65	T_{end}	-

H2	F69	T_{end}	-
L1	A36	T_{end}	+
H2	F76	T_{end}	-
M1	B24	T_{end}	+
H2	F8	T_{end}	-
L2	D10	T_{end}	+
H2	F80	T_{end}	-
M2	E42	T_{end}	+
H2	F90	T_{end}	-
H1	C10	T_{end}	+
VL2	H23	T_{end}	-
H2	F23	T_{end}	+
VL2	H30	T_{end}	-
VL2	H3	T_{end}	+
VL1	I3	T_{end}	-
VL1	I15	T_{end}	+
