# Supplementary figures and images for: Spontaneous whole-genome duplication restores fertility in interspecific hybrids
Source: Nat Commun. 2019 Sep 11;10:4126. doi: 10.1038/s41467-019-12041-8 (PMC6739354; doi:10.1038/s41467-019-12041-8)

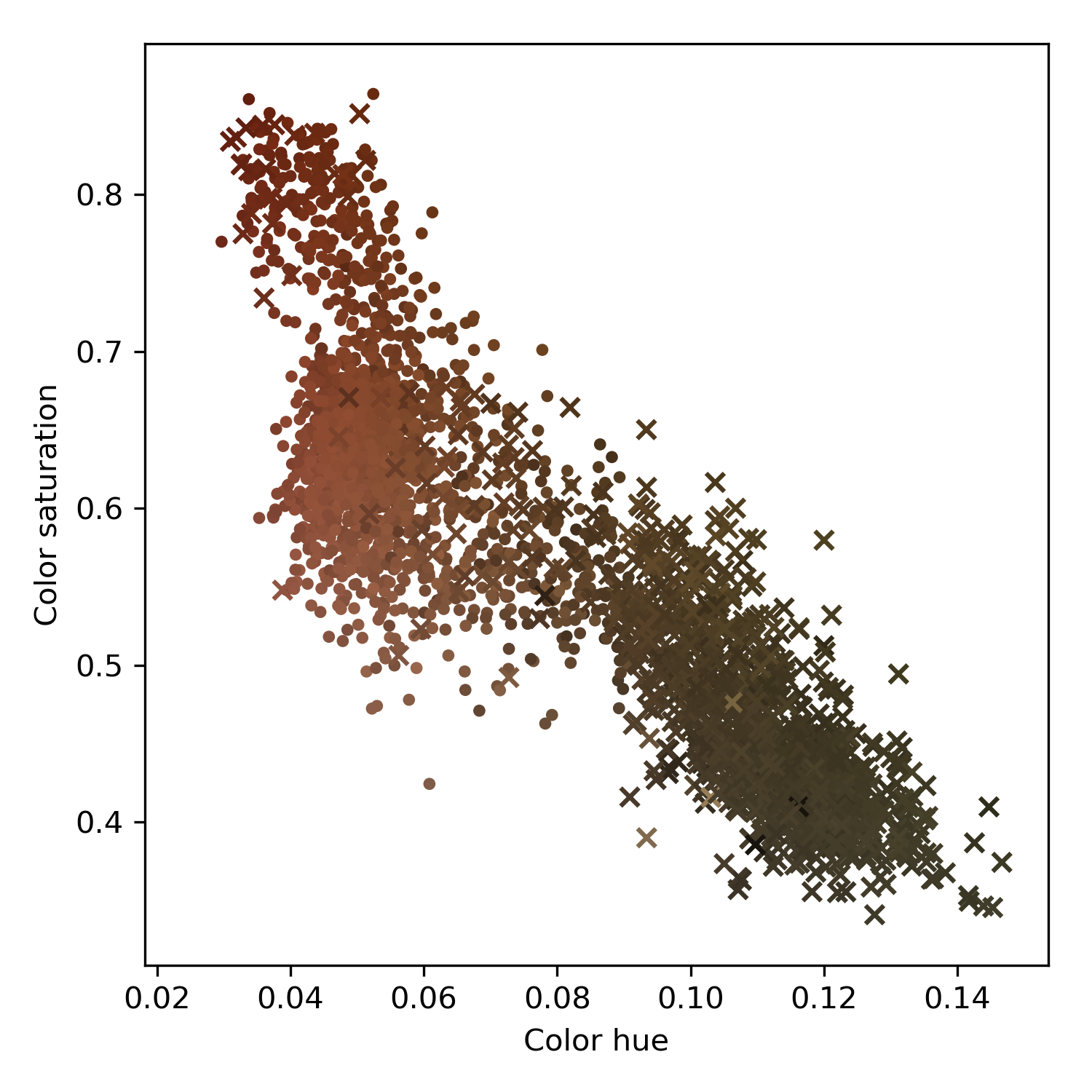

Supplement: Supplementary file 6 — Supplementary Data 2 [file 41467_2019_12041_MOESM6_ESM.zip › hue_sat.png]

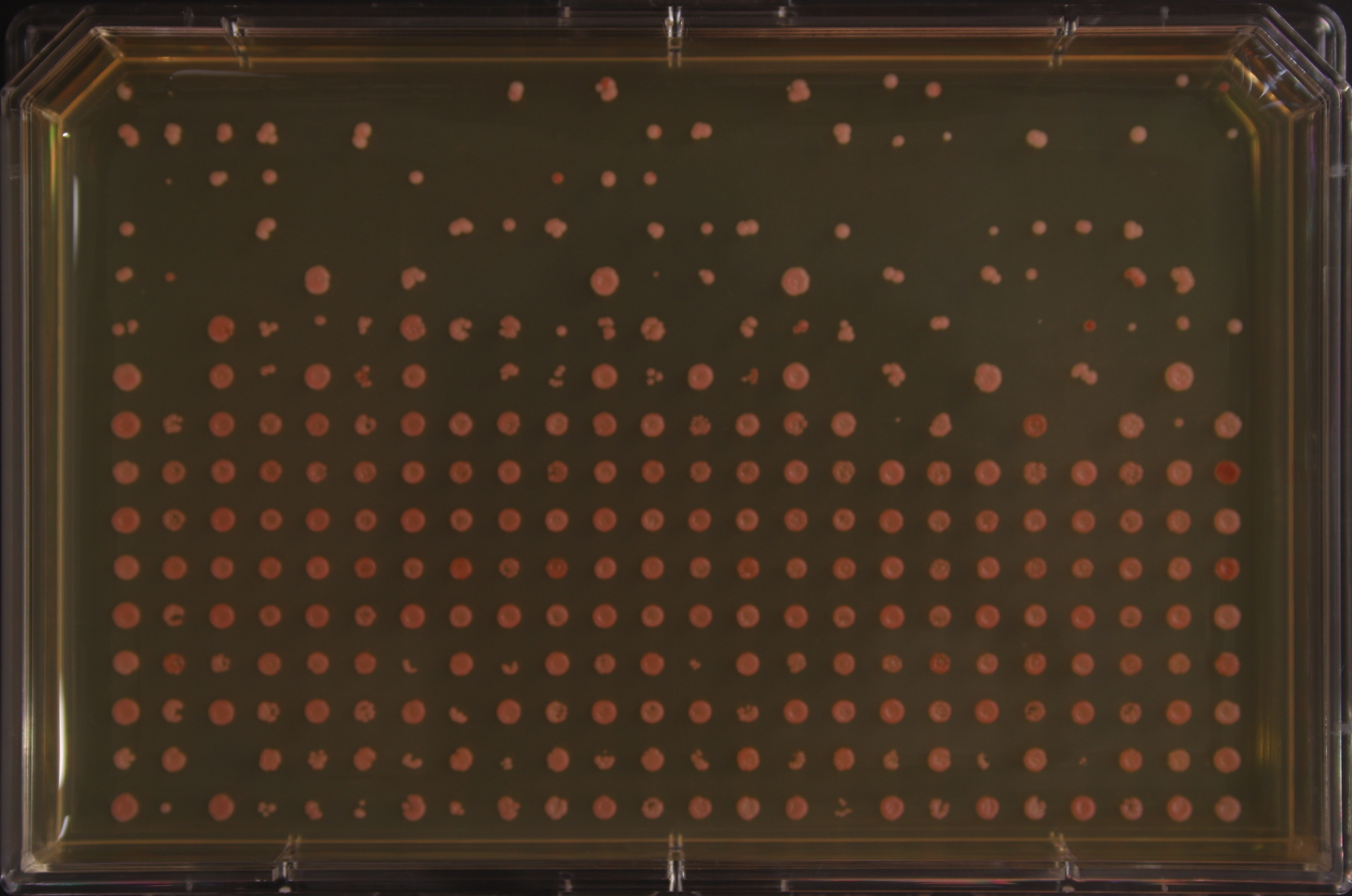

Supplement: Supplementary file 6 — Supplementary Data 2 [file 41467_2019_12041_MOESM6_ESM.zip › images/IMG_070_crop.JPG]

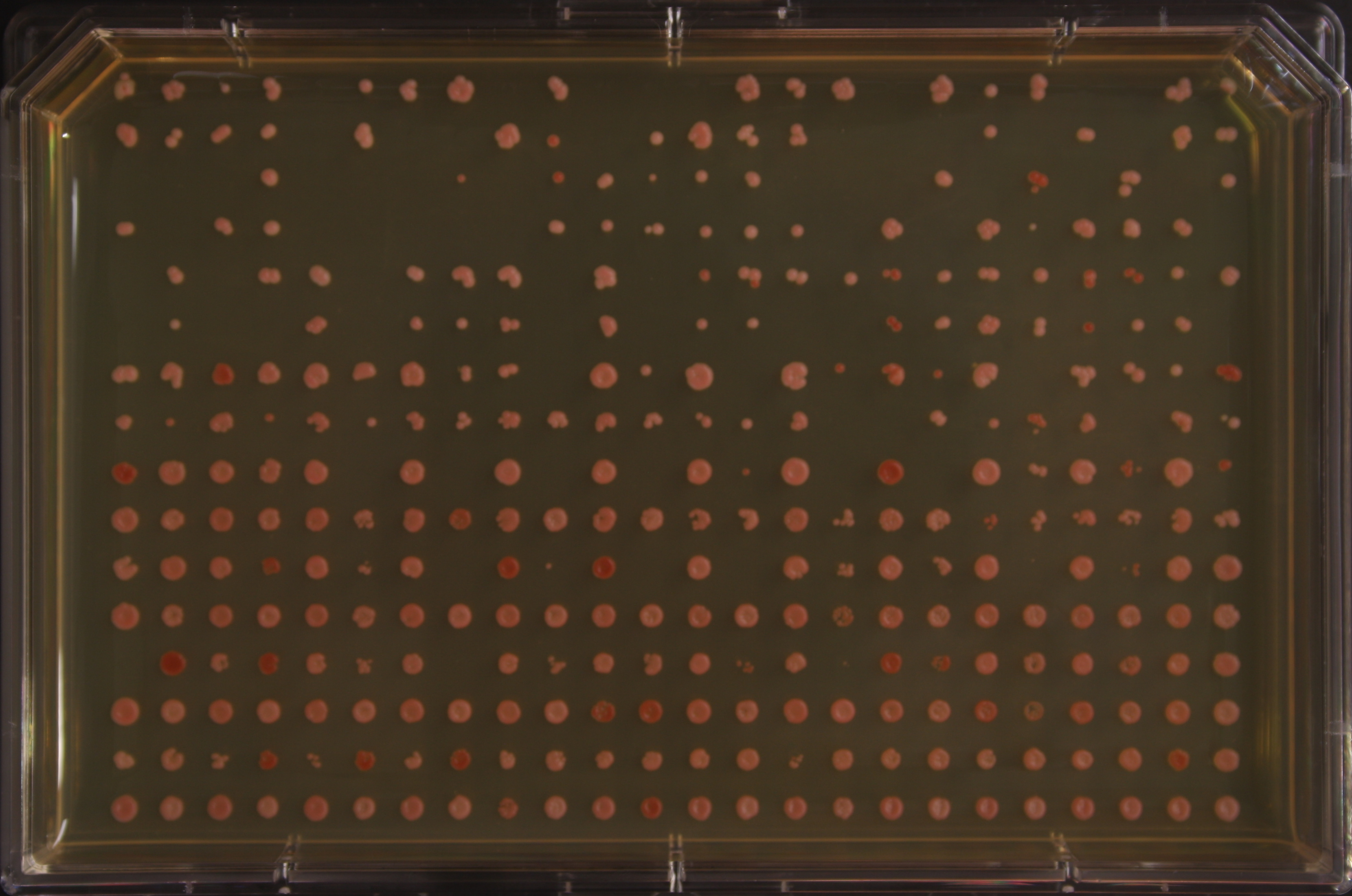

Supplement: Supplementary file 6 — Supplementary Data 2 [file 41467_2019_12041_MOESM6_ESM.zip › images/IMG_071_crop.JPG]

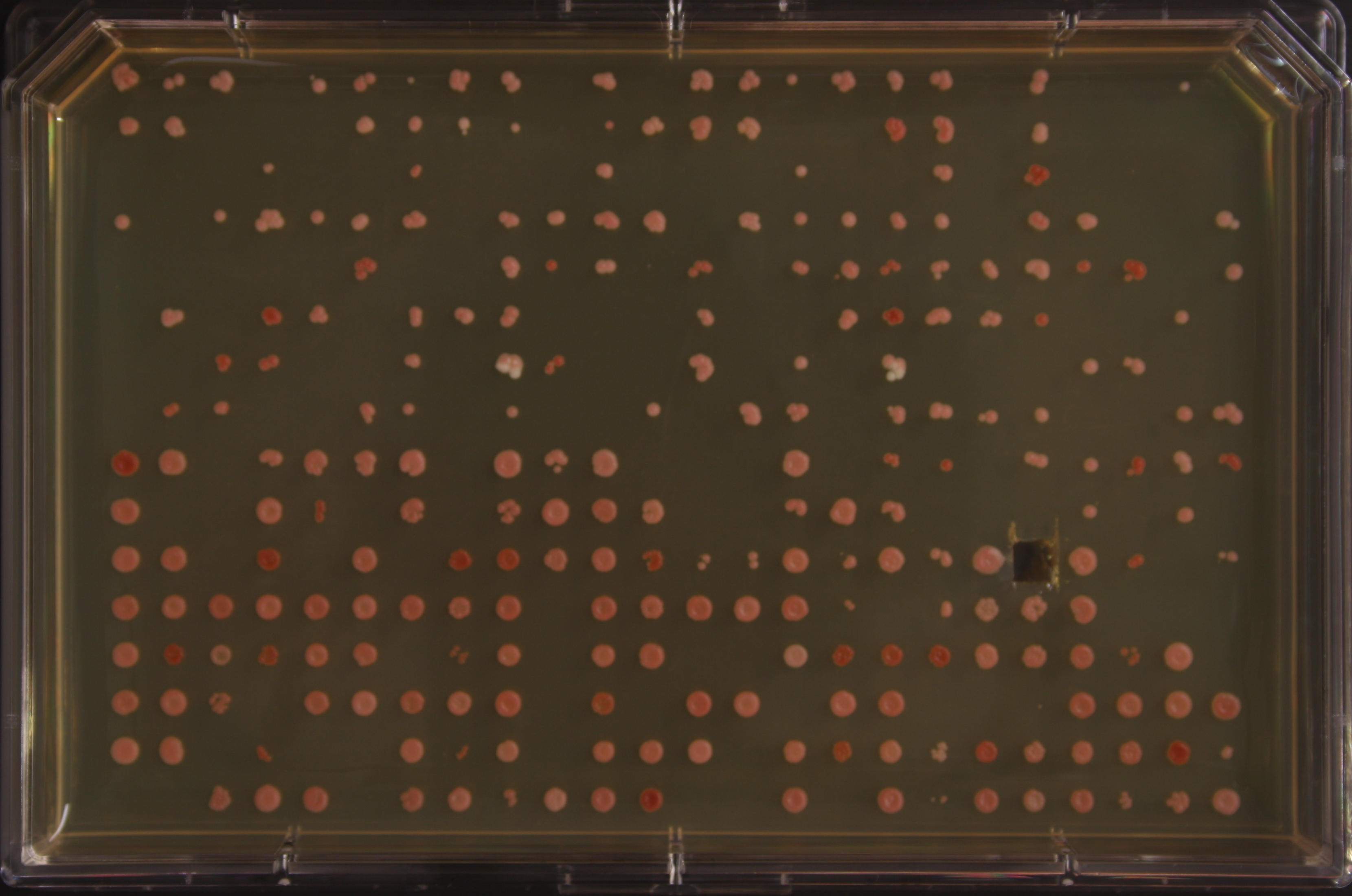

Supplement: Supplementary file 6 — Supplementary Data 2 [file 41467_2019_12041_MOESM6_ESM.zip › images/IMG_072_crop.JPG]

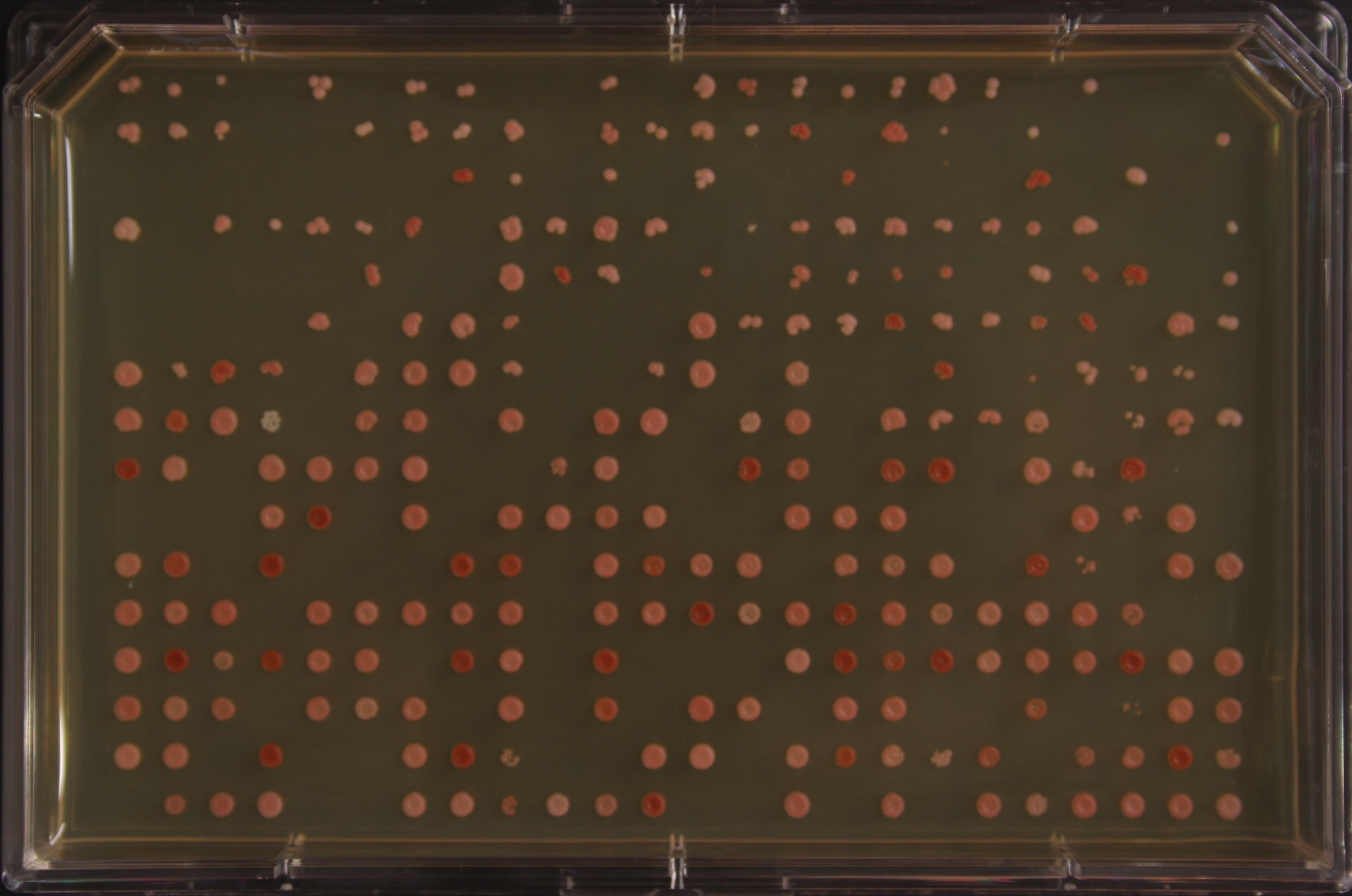

Supplement: Supplementary file 6 — Supplementary Data 2 [file 41467_2019_12041_MOESM6_ESM.zip › images/IMG_073_crop.JPG]

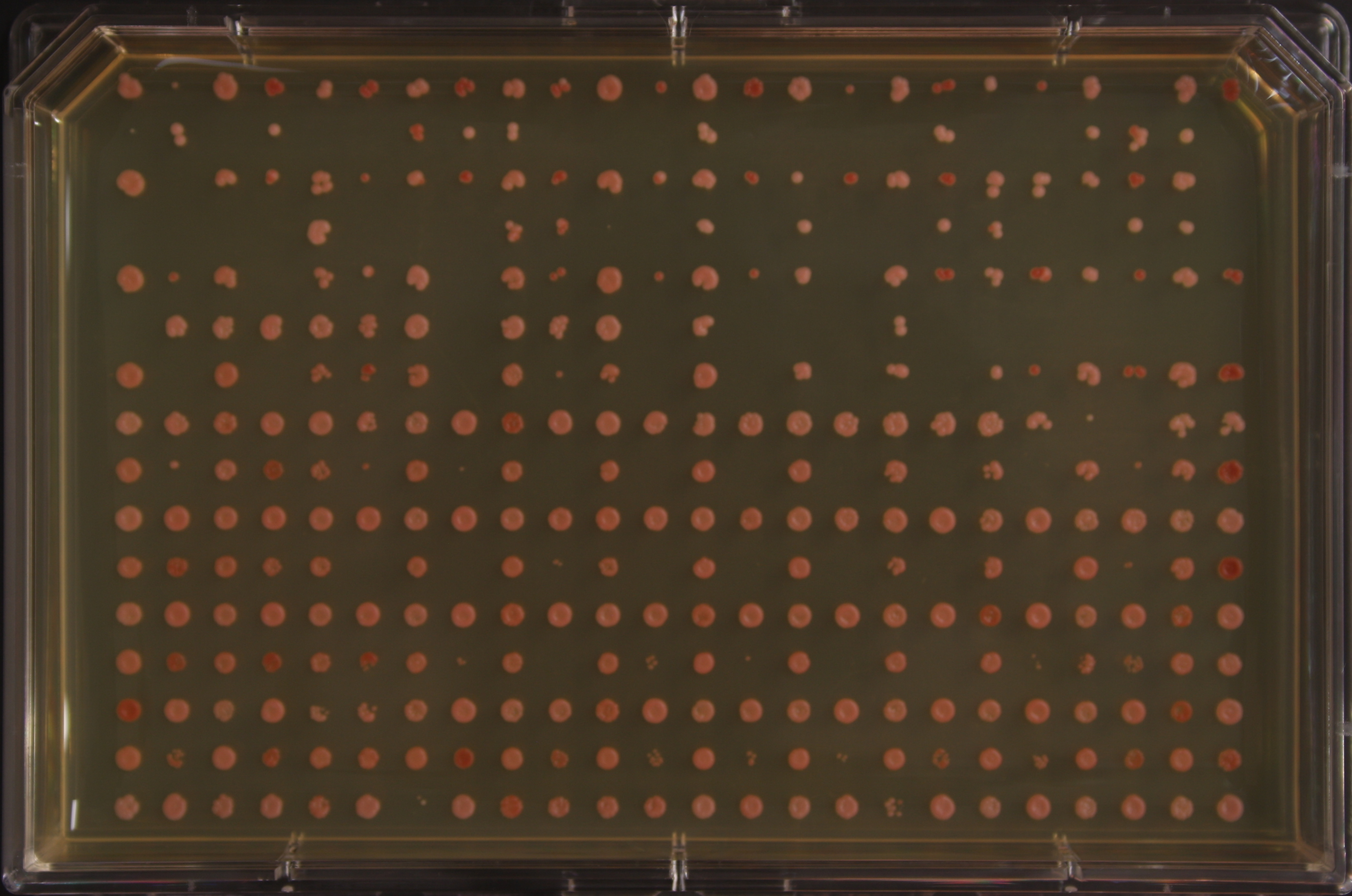

Supplement: Supplementary file 6 — Supplementary Data 2 [file 41467_2019_12041_MOESM6_ESM.zip › images/IMG_074_crop.JPG]

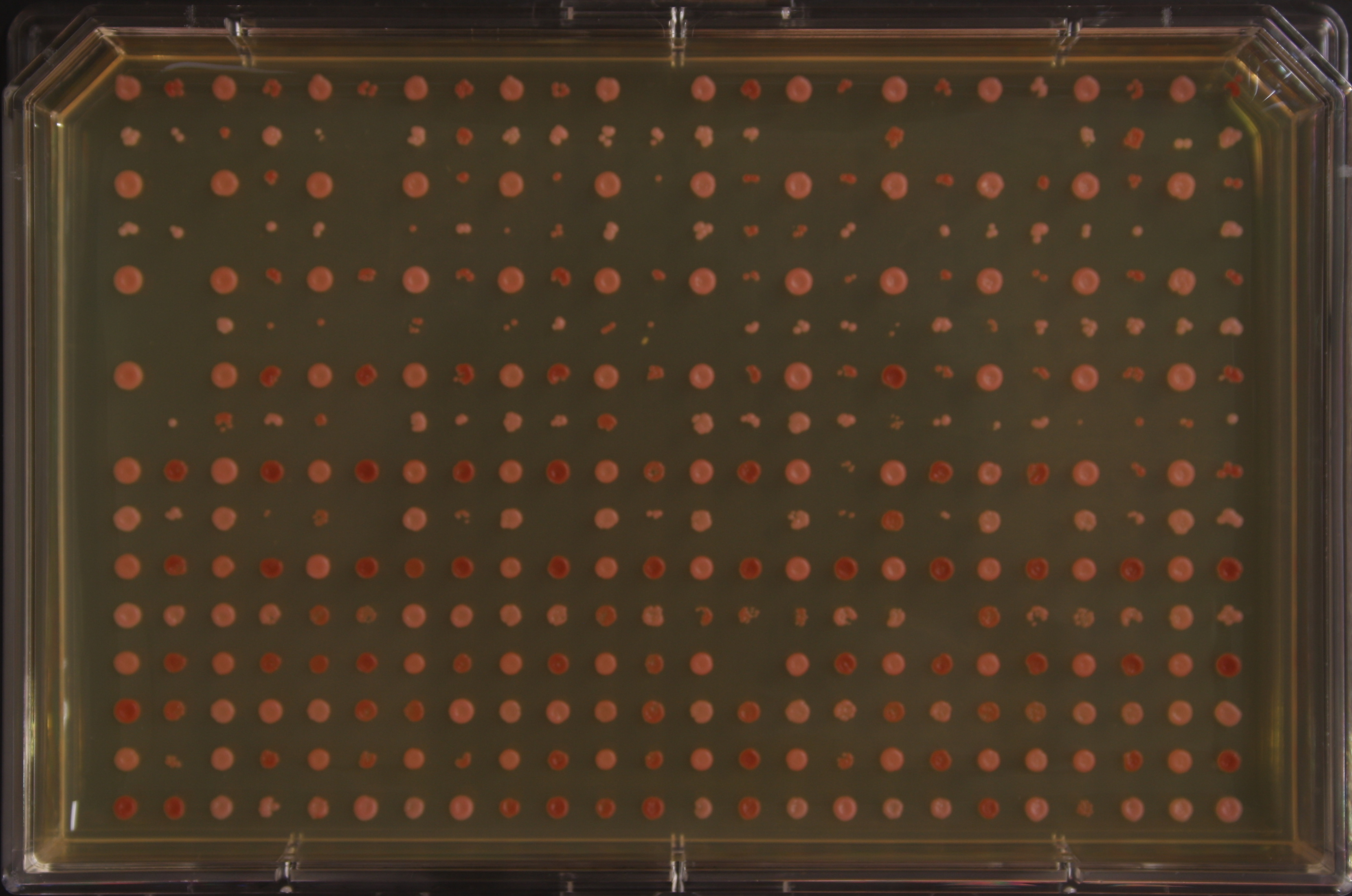

Supplement: Supplementary file 6 — Supplementary Data 2 [file 41467_2019_12041_MOESM6_ESM.zip › images/IMG_075_crop.JPG]

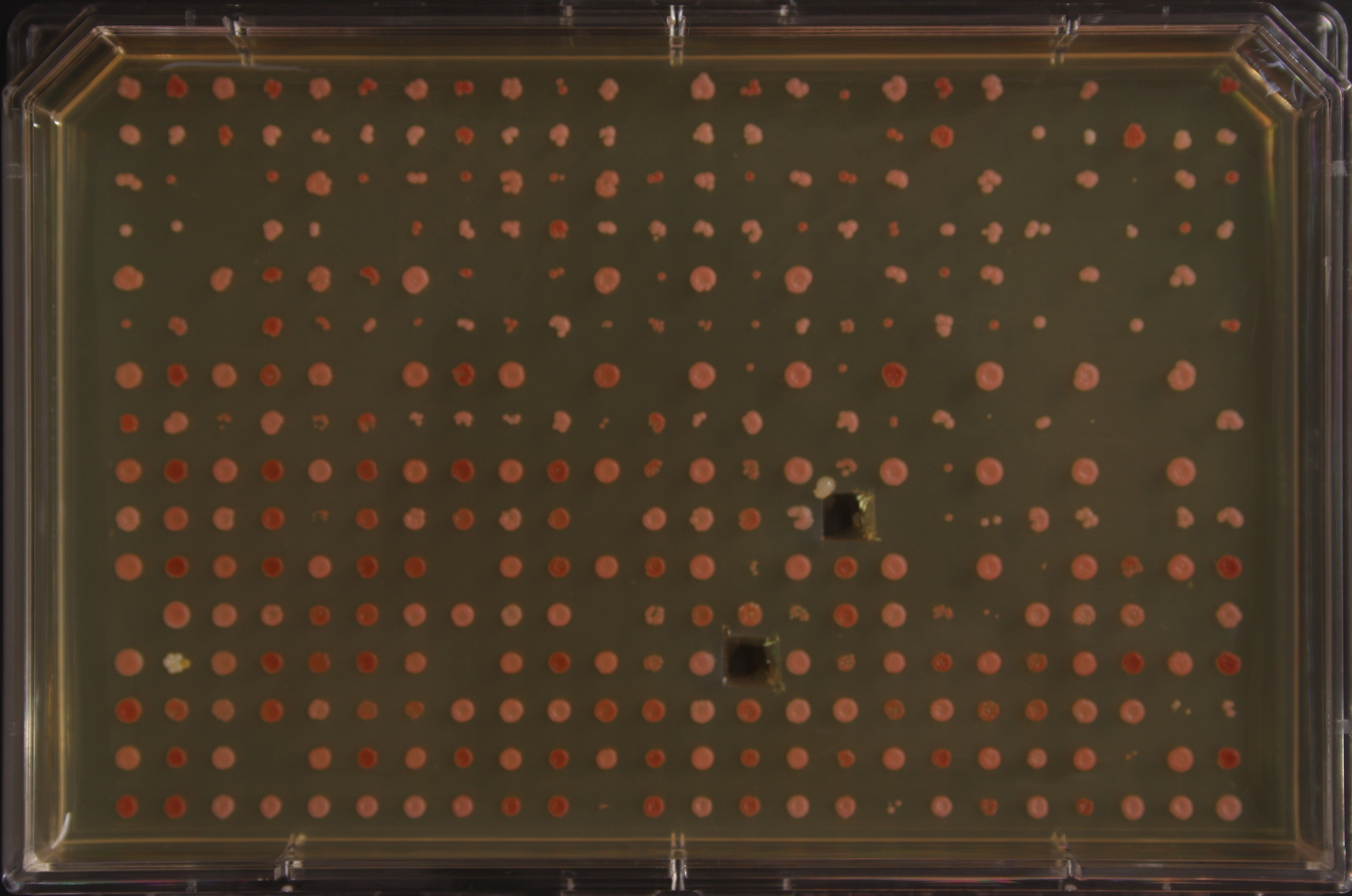

Supplement: Supplementary file 6 — Supplementary Data 2 [file 41467_2019_12041_MOESM6_ESM.zip › images/IMG_076_crop.JPG]

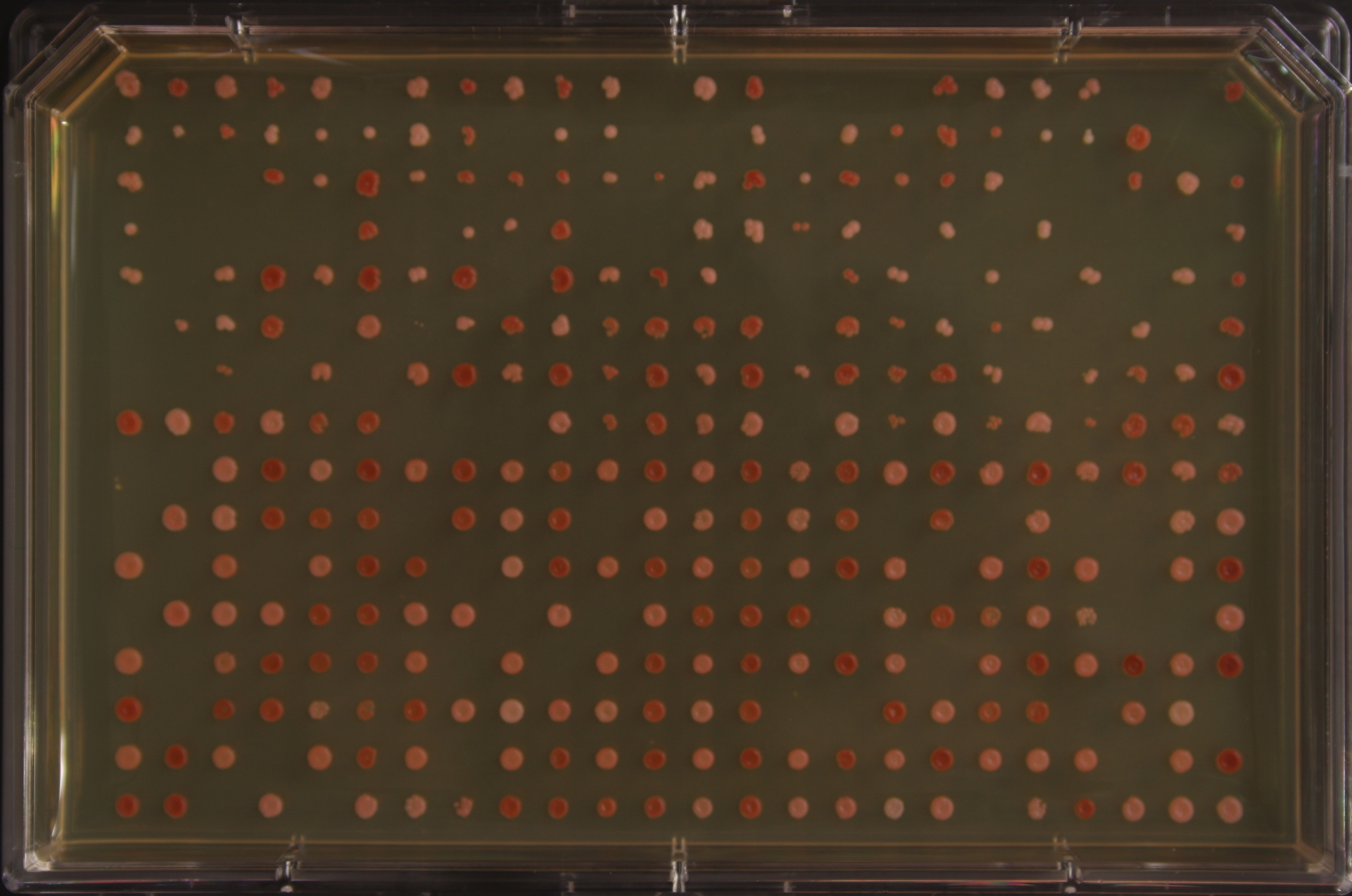

Supplement: Supplementary file 6 — Supplementary Data 2 [file 41467_2019_12041_MOESM6_ESM.zip › images/IMG_077_crop.JPG]

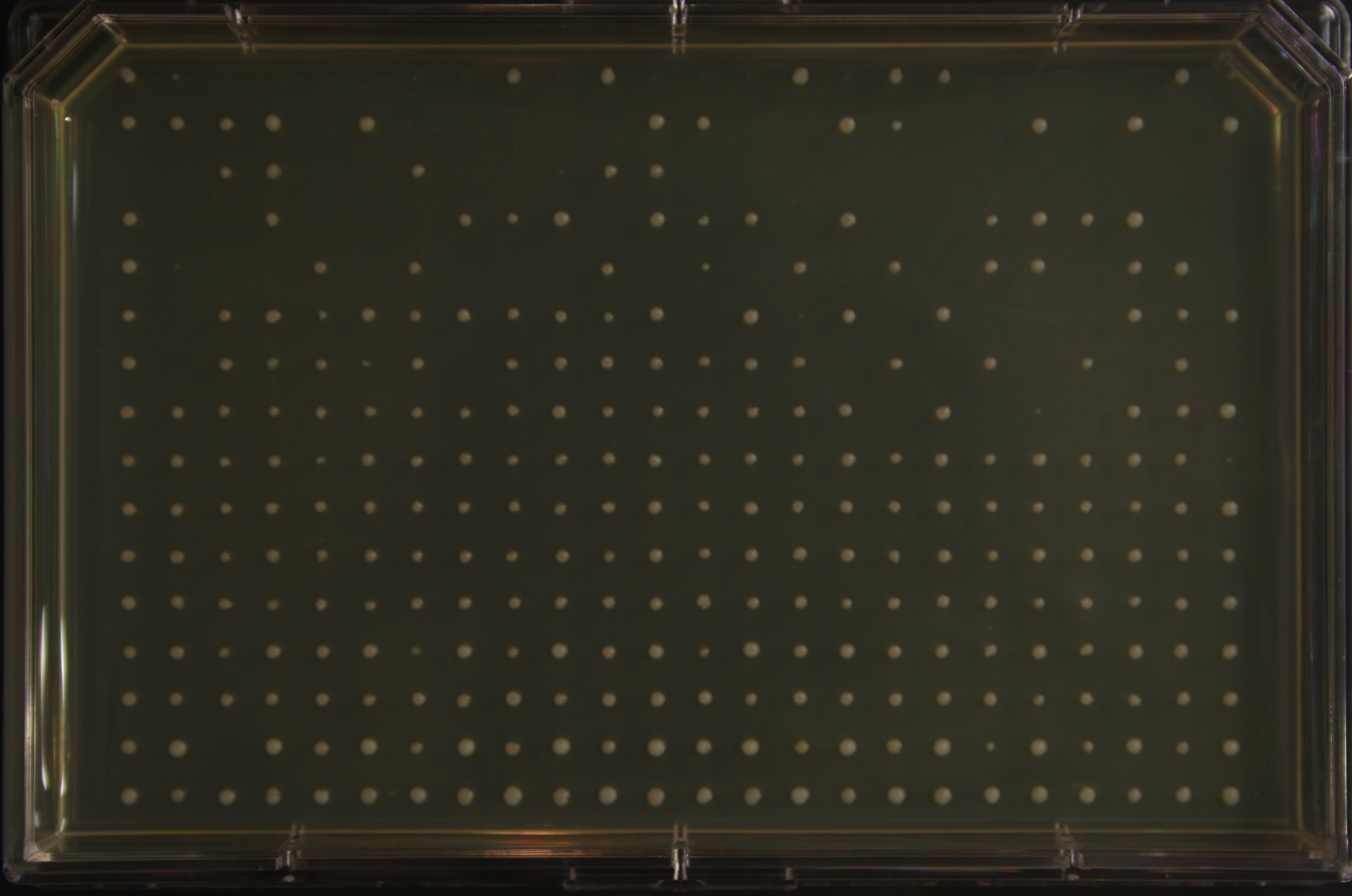

Supplement: Supplementary file 6 — Supplementary Data 2 [file 41467_2019_12041_MOESM6_ESM.zip › images/IMG_098_crop.JPG]

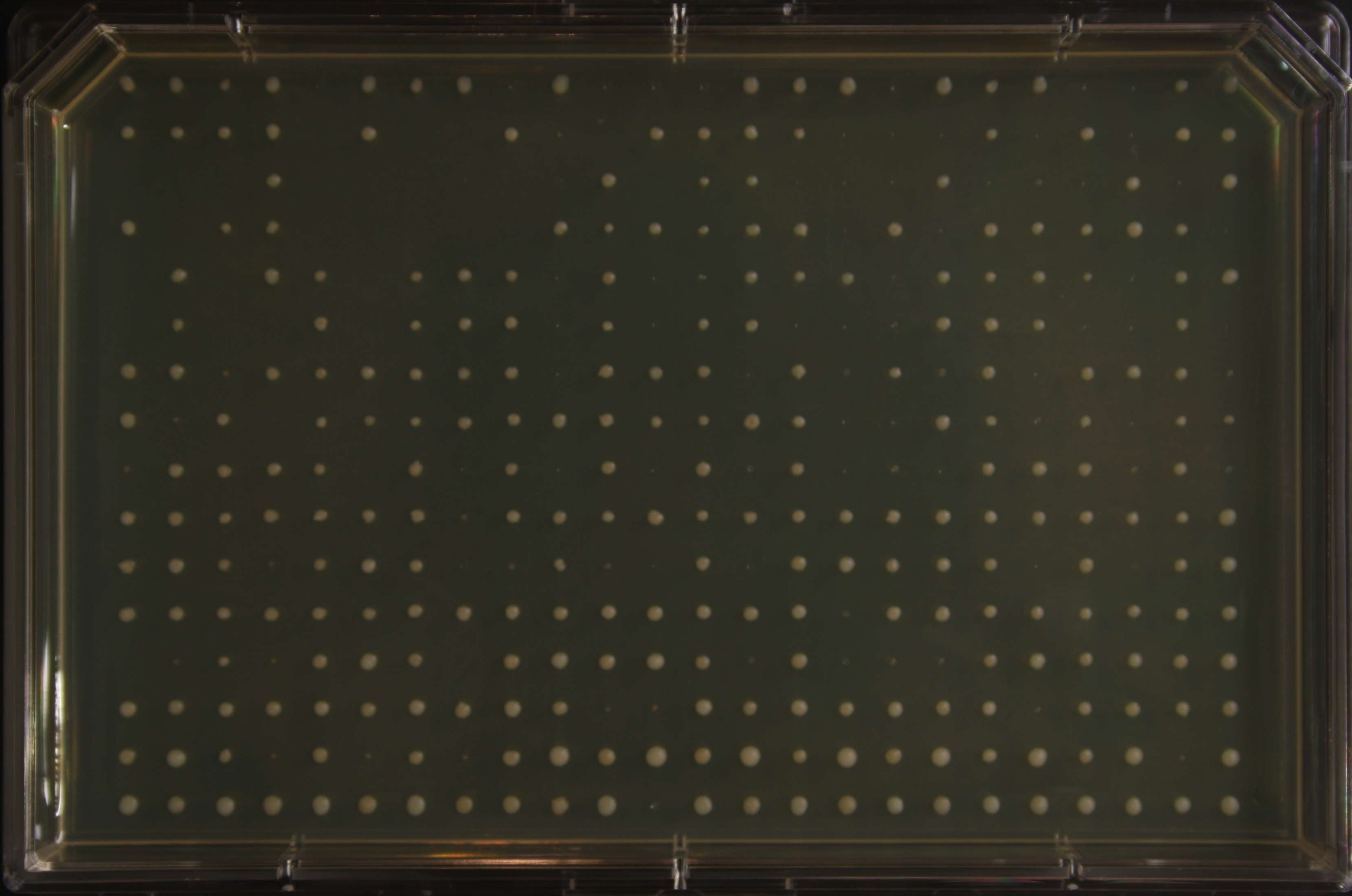

Supplement: Supplementary file 6 — Supplementary Data 2 [file 41467_2019_12041_MOESM6_ESM.zip › images/IMG_099_crop.JPG]

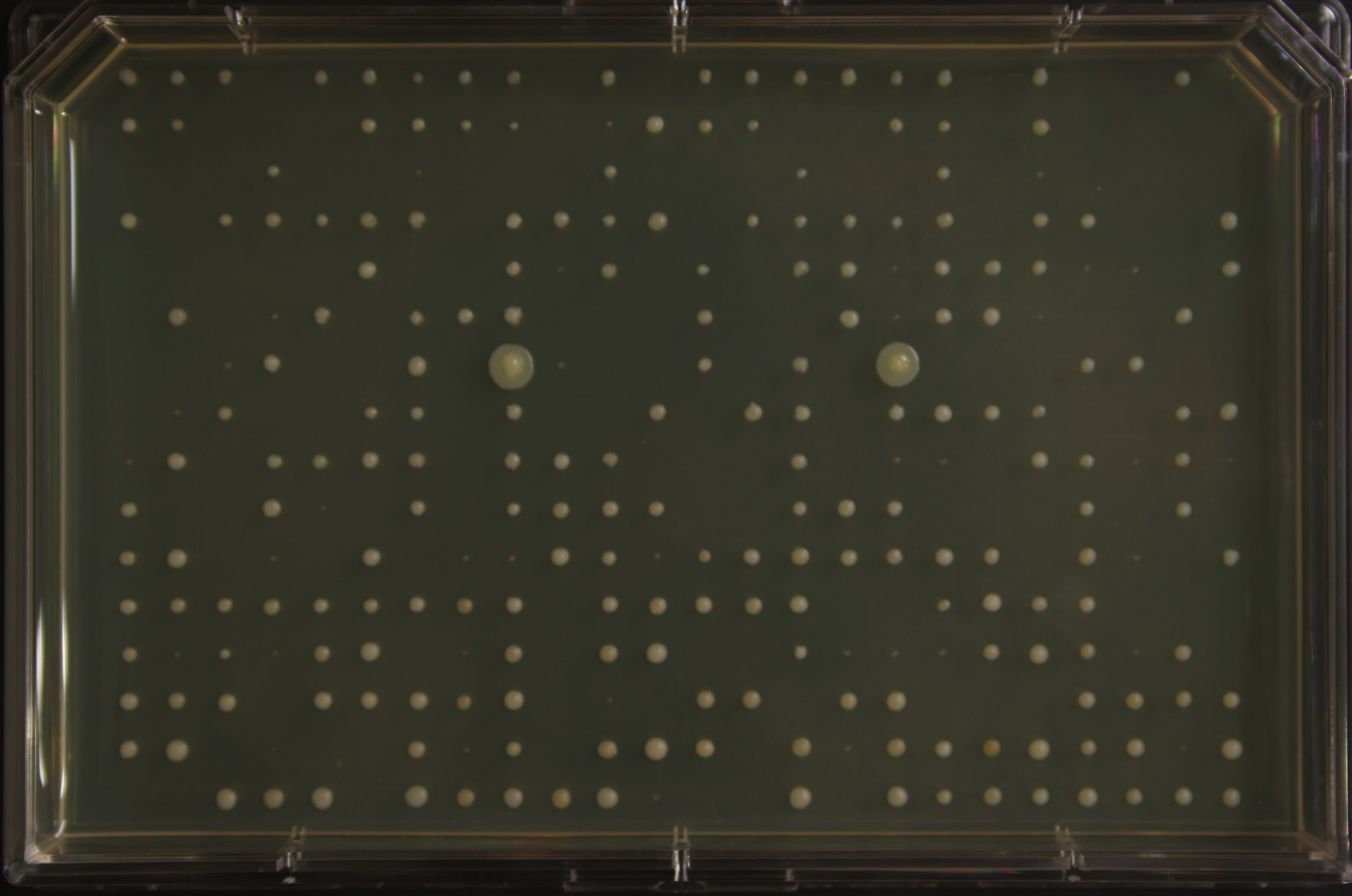

Supplement: Supplementary file 6 — Supplementary Data 2 [file 41467_2019_12041_MOESM6_ESM.zip › images/IMG_100_crop.JPG]

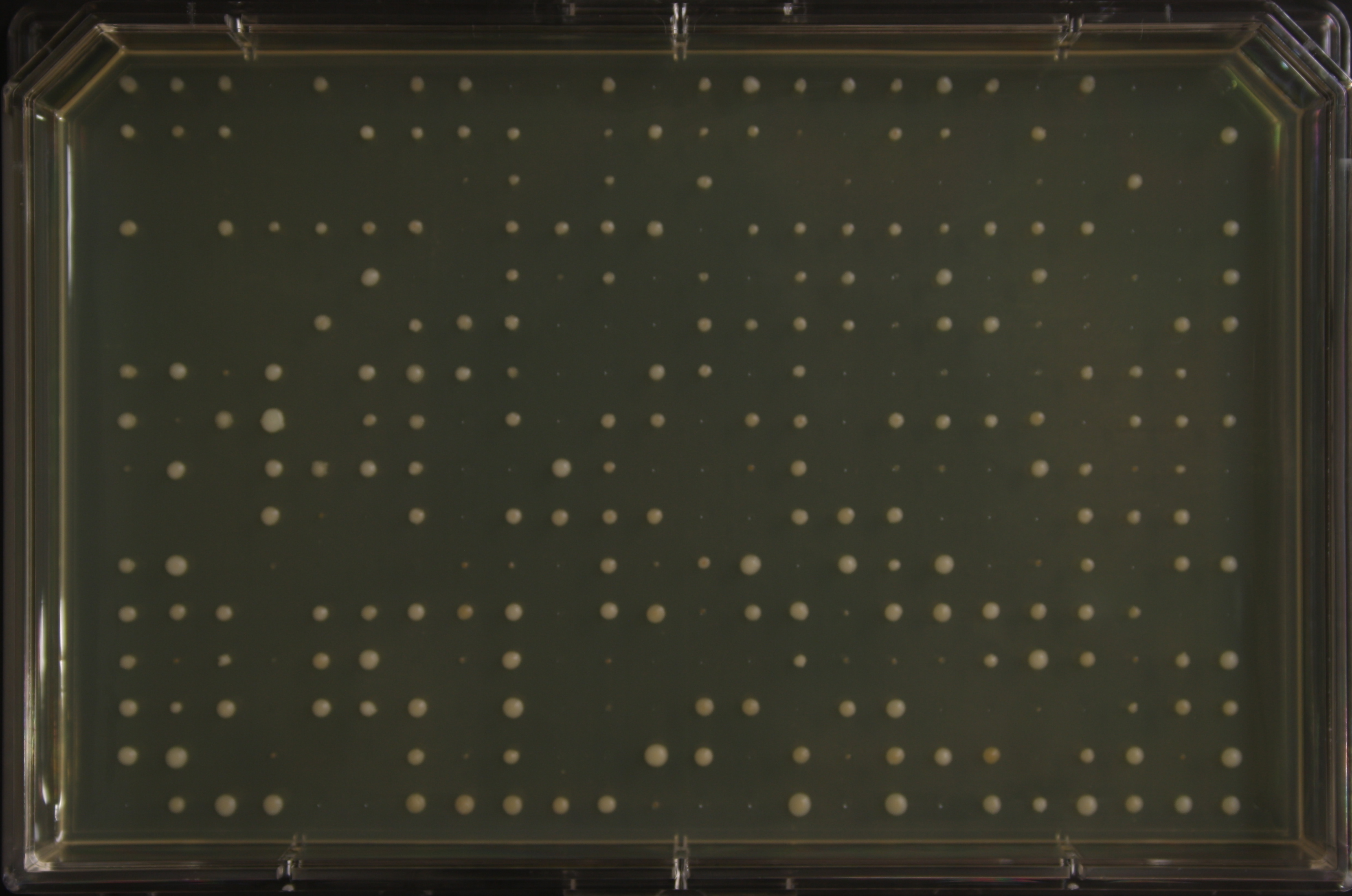

Supplement: Supplementary file 6 — Supplementary Data 2 [file 41467_2019_12041_MOESM6_ESM.zip › images/IMG_101_crop.JPG]

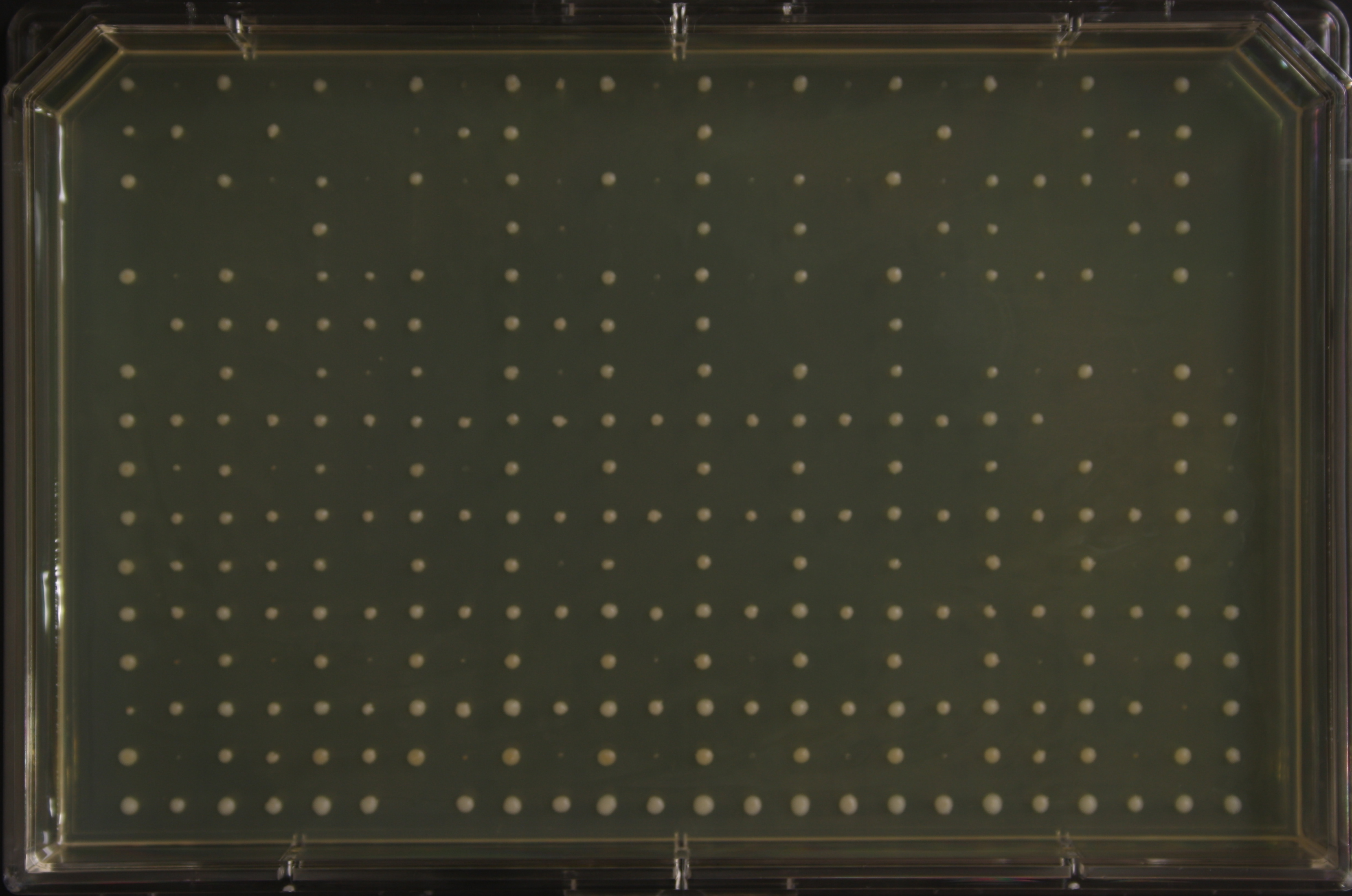

Supplement: Supplementary file 6 — Supplementary Data 2 [file 41467_2019_12041_MOESM6_ESM.zip › images/IMG_102_crop.JPG]

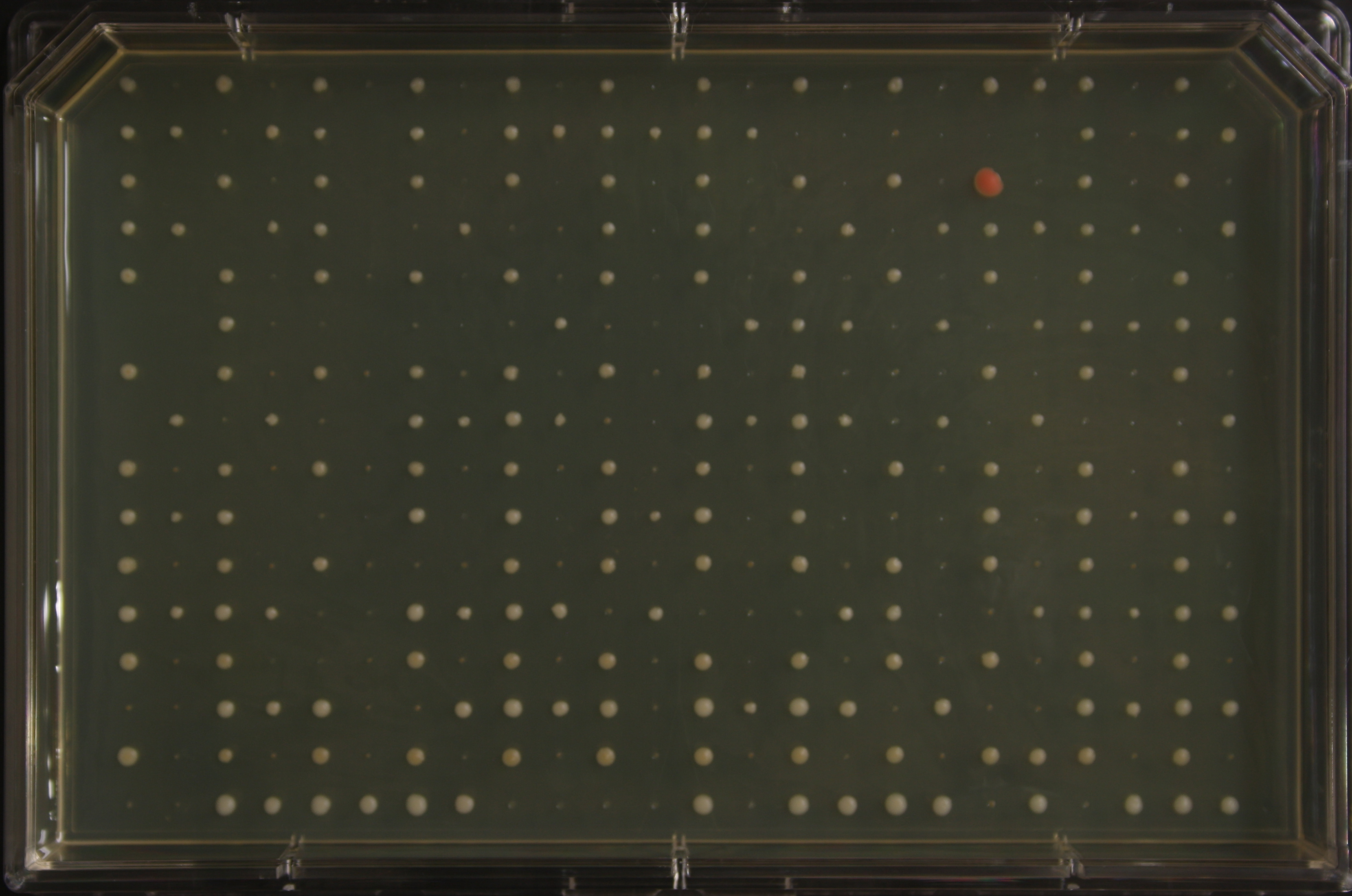

Supplement: Supplementary file 6 — Supplementary Data 2 [file 41467_2019_12041_MOESM6_ESM.zip › images/IMG_103_crop.JPG]

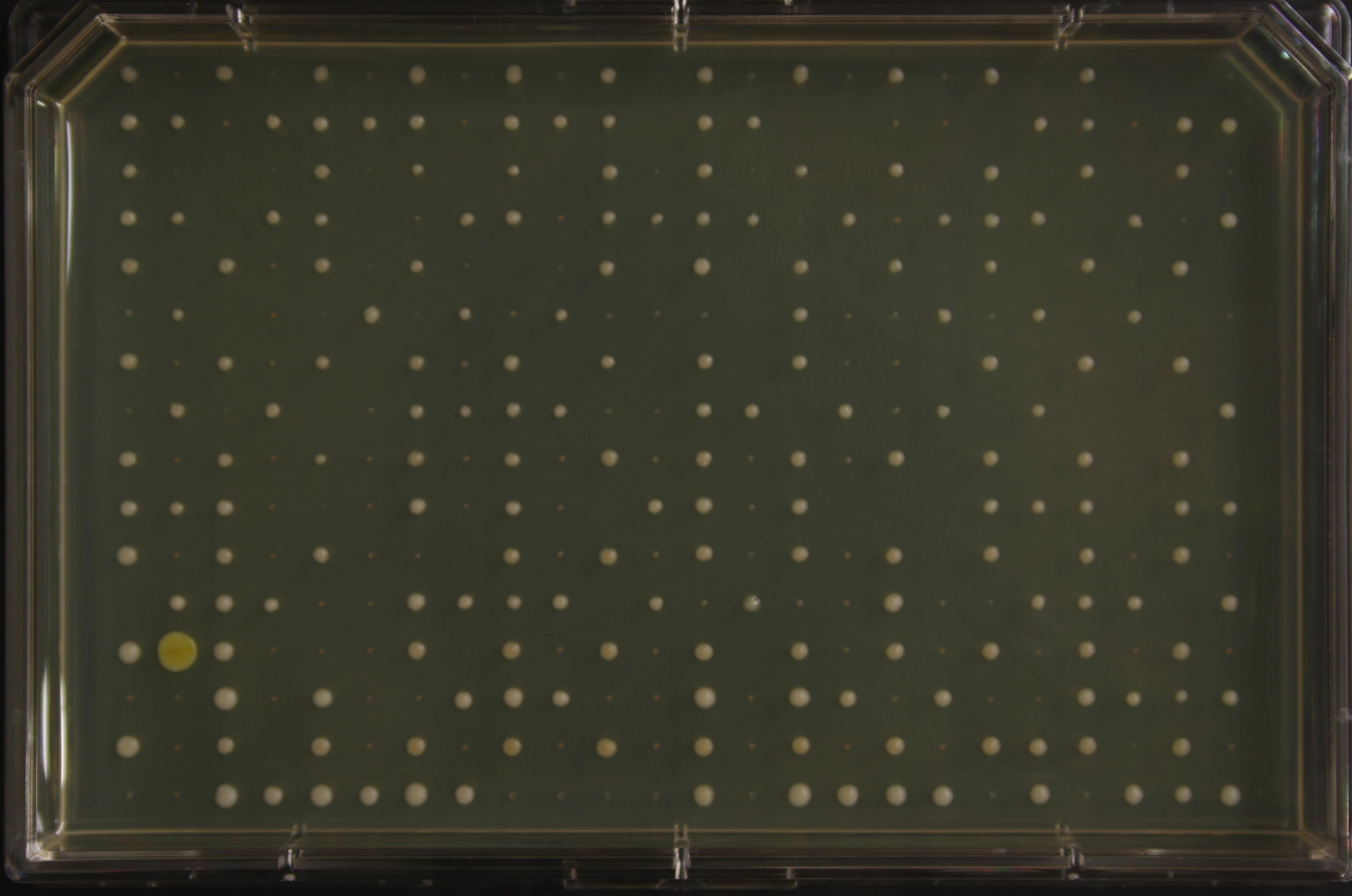

Supplement: Supplementary file 6 — Supplementary Data 2 [file 41467_2019_12041_MOESM6_ESM.zip › images/IMG_104_crop.JPG]

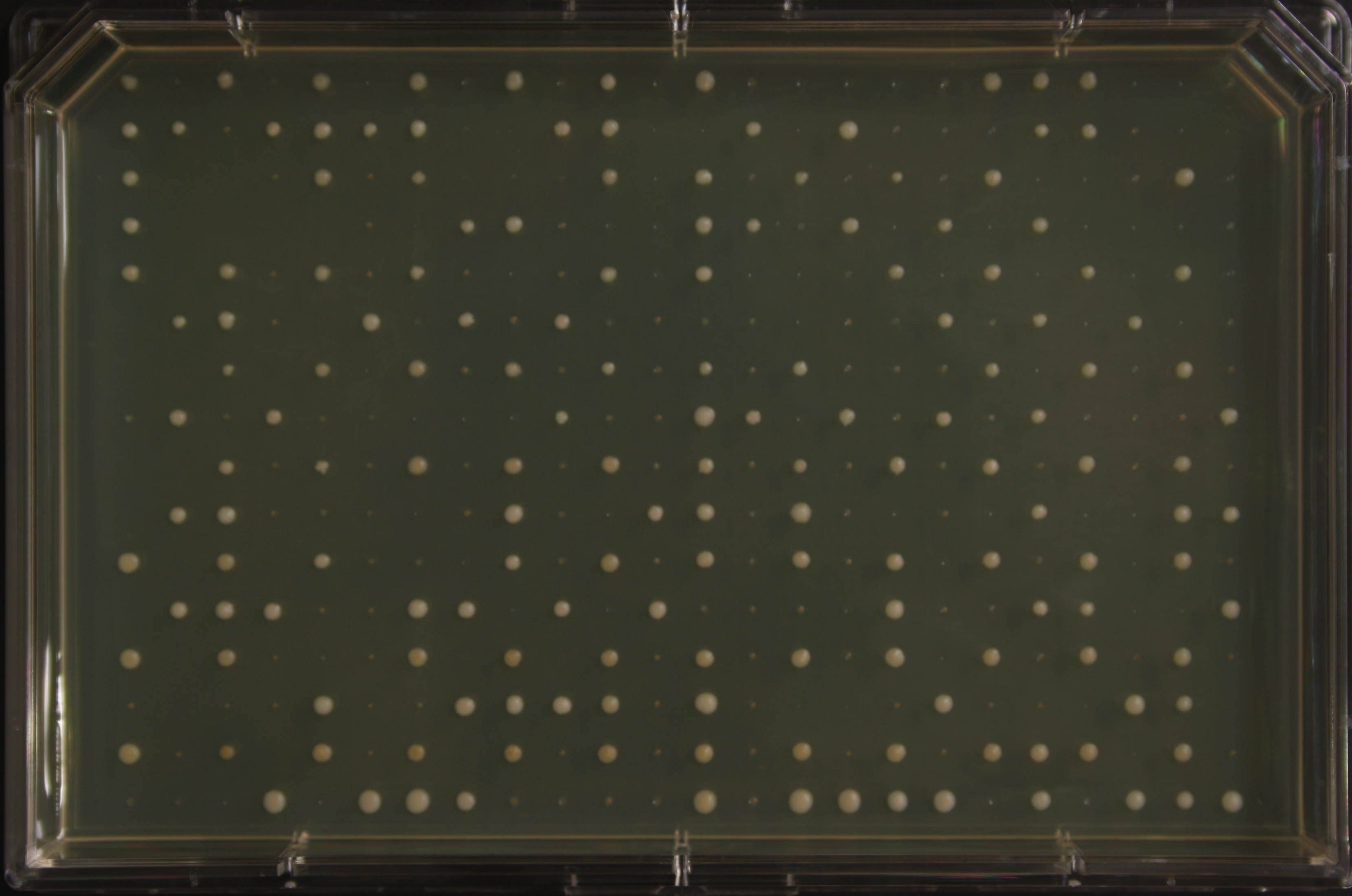

Supplement: Supplementary file 6 — Supplementary Data 2 [file 41467_2019_12041_MOESM6_ESM.zip › images/IMG_105_crop.JPG]

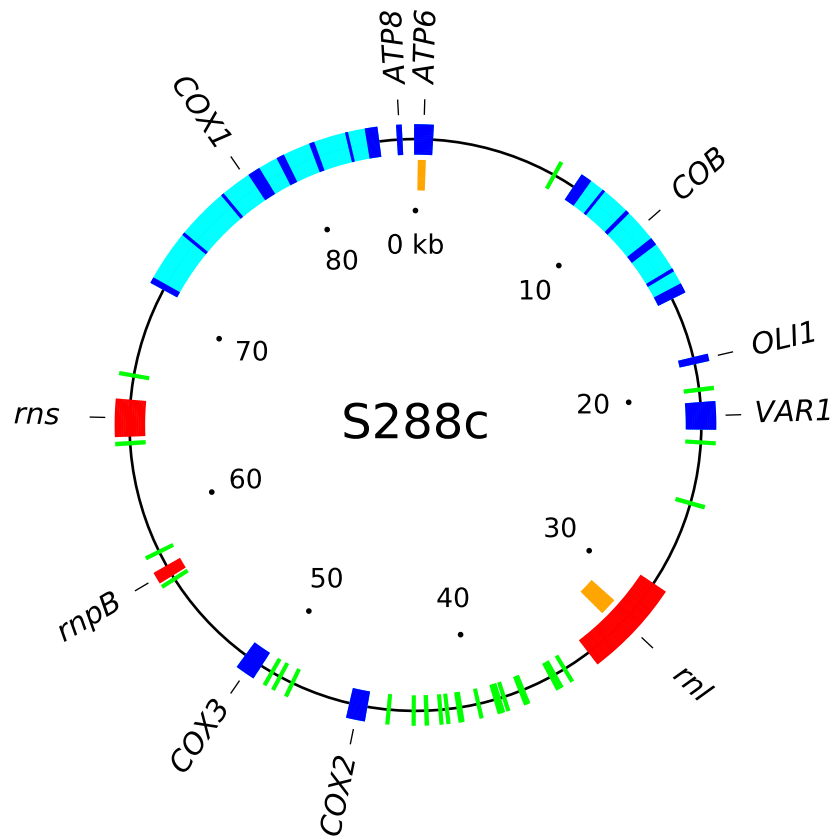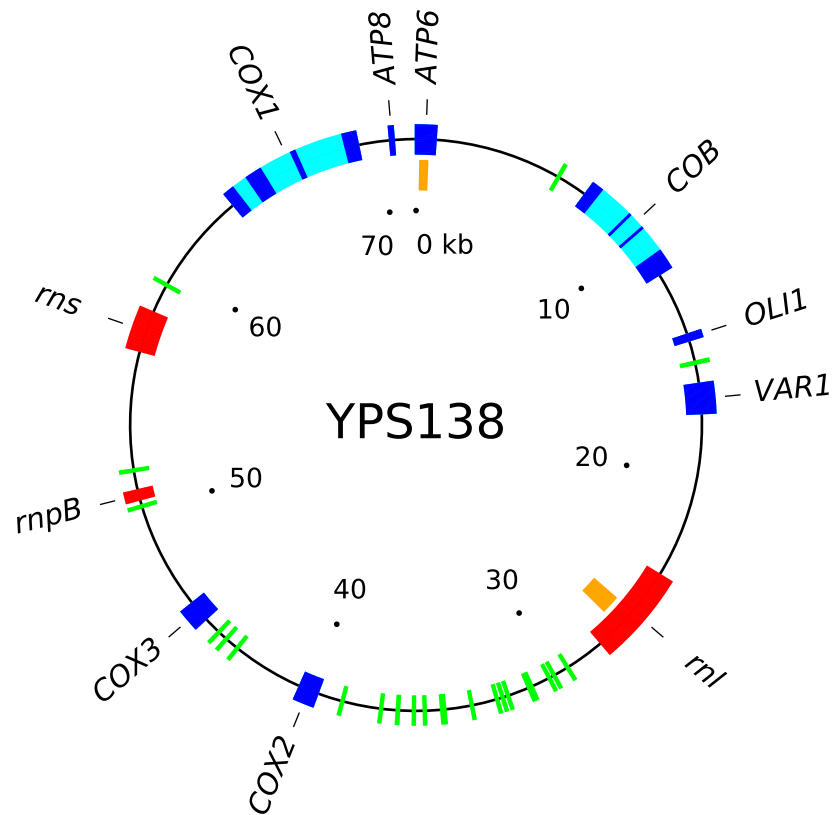

Supplement: Supplementary file 6 — Supplementary Data 2 [file 41467_2019_12041_MOESM6_ESM.zip › mito_map_5x10.pdf]

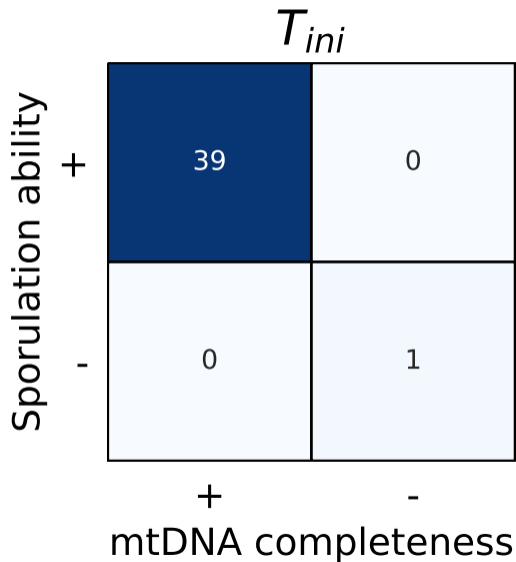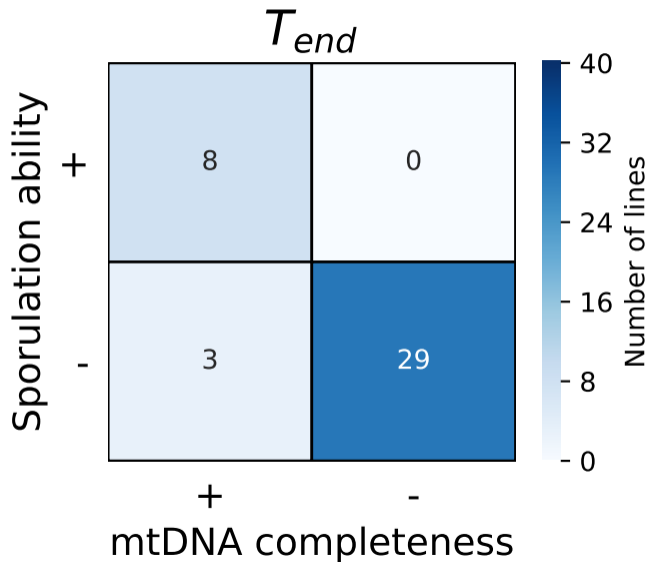

Supplement: Supplementary file 6 — Supplementary Data 2 [file 41467_2019_12041_MOESM6_ESM.zip › mtDNAcomplete_sporulation.pdf]

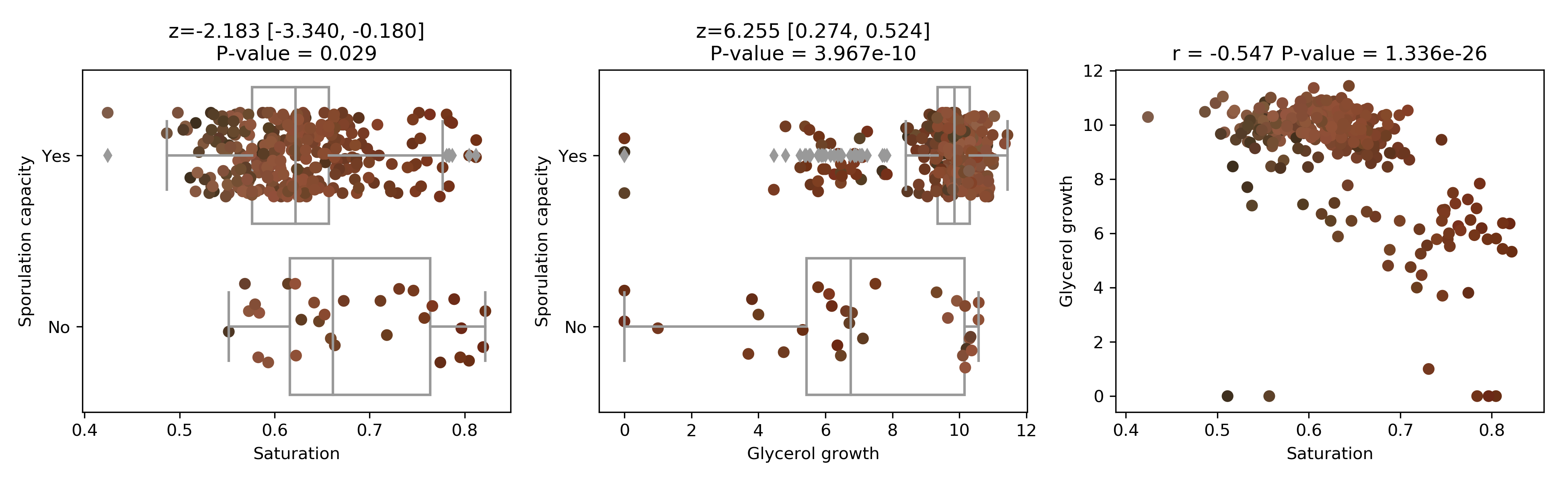

Supplement: Supplementary file 6 — Supplementary Data 2 [file 41467_2019_12041_MOESM6_ESM.zip › spo_ability_sat_glyc.png]

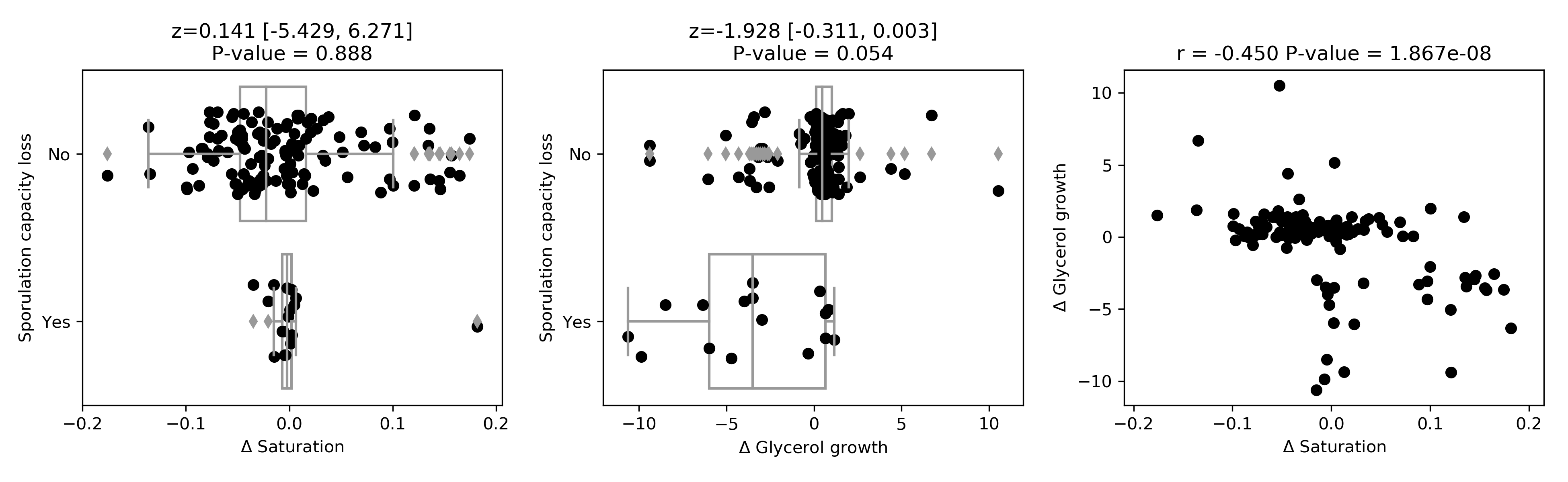

Supplement: Supplementary file 6 — Supplementary Data 2 [file 41467_2019_12041_MOESM6_ESM.zip › spo_loss_sat_glyc_diff.png]
